# Perception without preconception: comparison between the human and machine learner in recognition of tissues from histological sections

**DOI:** 10.1038/s41598-022-20012-1

**Published:** 2022-09-30

**Authors:** Sanghita Barui, Parikshit Sanyal, K. S. Rajmohan, Ajay Malik, Sharmila Dudani

**Affiliations:** 1 Department of Pathology, Base Hospital Delhi, New Delhi, 110010 India; 2 AHRR Delhi, Dhaula Kuan, New Delhi, 110010 India; 3 Lab Director & Head, Dept of Pathology, Yashoda Hospital & Research Centre, Nehru Nagar, Ghaziabad, India; 4 Department of Pathology, ACMS Delhi, Brar Square, New Delhi, 110010 India

**Keywords:** Computational biology and bioinformatics, Cells

## Abstract

Deep neural networks (DNNs) have shown success in image classification, with high accuracy in recognition of everyday objects. Performance of DNNs has traditionally been measured assuming human accuracy is perfect. In specific problem domains, however, human accuracy is less than perfect and a comparison between humans and machine learning (ML) models can be performed. In recognising everyday objects, humans have the advantage of a lifetime of experience, whereas DNN models are trained only with a limited image dataset. We have tried to compare performance of human learners and two DNN models on an image dataset which is novel to both, i.e. histological images. We thus aim to eliminate the advantage of prior experience that humans have over DNN models in image classification. Ten classes of tissues were randomly selected from the undergraduate first year histology curriculum of a Medical School in North India. Two machine learning (ML) models were developed based on the VGG16 (VML) and Inception V2 (IML) DNNs, using transfer learning, to produce a 10-class classifier. One thousand (1000) images belonging to the ten classes (i.e. 100 images from each class) were split into training (700) and validation (300) sets. After training, the VML and IML model achieved 85.67 and 89% accuracy on the validation set, respectively. The training set was also circulated to medical students (MS) of the college for a week. An online quiz, consisting of a random selection of 100 images from the validation set, was conducted on students (after obtaining informed consent) who volunteered for the study. 66 students participated in the quiz, providing 6557 responses. In addition, we prepared a set of 10 images which belonged to different classes of tissue, *not present in training set* (i.e. out of training scope or OTS images). A second quiz was conducted on medical students with OTS images, and the ML models were also run on these OTS images. The overall accuracy of MS in the first quiz was 55.14%. The two ML models were also run on the first quiz questionnaire, producing accuracy between 91 and 93%. The ML models scored more than 80% of medical students. Analysis of confusion matrices of both ML models and all medical students showed dissimilar error profiles. However, when comparing the subset of students who achieved similar accuracy as the ML models, the error profile was also similar. Recognition of ‘stomach’ proved difficult for both humans and ML models. In 04 images in the first quiz set, both VML model and medical students produced highly equivocal responses. Within these images, a pattern of bias was uncovered–the tendency of medical students to misclassify ‘liver’ tissue. The ‘stomach’ class proved most difficult for both MS and VML, producing 34.84% of all errors of MS, and 41.17% of all errors of VML model; however, the IML model committed most errors in recognising the ‘skin’ class (27.5% of all errors). Analysis of the convolution layers of the DNN outlined features in the original image which might have led to misclassification by the VML model. In OTS images, however, the medical students produced better overall score than both ML models, i.e. they successfully recognised patterns of similarity between tissues and could generalise their training to a novel dataset. Our findings suggest that within the scope of training, ML models perform better than 80% medical students with a distinct error profile. However, students who have reached accuracy close to the ML models, tend to replicate the error profile as that of the ML models. This suggests a degree of similarity between how machines and humans extract features from an image. If asked to recognise images outside the scope of training, humans perform better at recognising patterns and likeness between tissues. This suggests that ‘training’ is not the same as ‘learning’, and humans can extend their pattern-based learning to different domains outside of the training set.

## Introduction

Deep Neural Networks (DNN) have emerged as capable models for visual recognition; in fact, some have suggested that they are *in-silico* models of the human visual system^1^. The success of DNNs in recognition of everyday objects^2^ has raised speculations that activations in the intermediate layers of DNNs reflect the neural activity in visual cortex^3^. Such conclusions have often been drawn from observing DNN models that closely match human performance in recognition of everyday objects (cars, books, plants, animals, fruits etc.), i.e. those objects that the non-expert human is likely to encounter in his day-to-day life^4^. In some specialised domains, DNN models match or outperform humans: for example, there exist DNN models trained to perform a binary classification—‘tuberculosis’ or ‘healthy’—from chest radiographs^5^. However, such specialised models are of limited scope and do not generalise to the larger problem of visual recognition of random objects.

Since last decade, several DNNs have been developed which have been trained with huge image datasets, such as ImageNet^6^, and can recognise a large class of everyday objects, i.e. they can function as generalised object identifiers^7^. However, such DNNs are not free from errors^8^, and such errors offer a window into the inner mechanisms of the model. This presents an opportunity to compare DNN models with humans. Research on differences between machine and human perception has been carried out on macroscopic, everyday objects (both with normal and low intensity signals)^9^, gestures and motions^10^, and specially prepared image datasets for ease of comparison^11^. In some of these test conditions, such as noisy and distorted images, humans perform significantly better^12^. However, any such comparison is bound to suffer from a degree of bias, because human brains are trained for visual recognition since birth – and typically, recognition of everyday objects/symbols by a human is instantaneous, requiring almost no cognitive action. Machine learning models, however advanced, are trained on finite datasets that can never match the breadth and depth of the human visual experience. Thus, they are subject to several shortcomings: for example, a special class of images which have been subtly modified from the original, and which the human can discern quite easily, can often produce a drastically wrong result from a well trained DNN model. Such ‘adversarial’ images are increasingly becoming relevant in diverse domains such as cyber security and healthcare^13^. In addition, DNN models may classify a completely random array of pixels as a real-life object^14^, which raises speculations whether DNNs ‘perceive’ objects in the conventional sense of the word.

Pure perception, unbiased by prior knowledge, has been defined by several philosophic schools. In the *Nyaya-sutra*, an early philosophic text from India, the author Gautama mentions: “Perception is a cognition which arises from the contact of the sense organ and object and is not impregnated by words, is unerring, and well-ascertained” ^15^. Implicit in such a definition is that there should be no prior memory (‘word’) of the perceived object, in the ‘agent’ who perceives. The *agent*, in this case, might be a human or a machine learning (ML) model. Thus, any comparison between human and machine learning is bound to be affected by the bias of antedate, i.e. the prior experience of humans in recognition of everyday objects. We felt that to compare the performance of humans and DNN, a novel dataset, which is new to both humans and machines, is required.

It is in this under-explored niche that histological image recognition finds its use. Typically, human beings are not trained to recognise tissue type from histological images by default; only a very select subset of humans (medical students, zoologists) will ever learn how to recognise tissue type from histological images. And this presents a window of opportunity for comparison of human and machine learning, because both the machine and the human have had no prior experience in classifying histological images.

### Brief review of relevant literature

The earliest precursor of DNNs is the Neocognitron developed by Fukushima et al. in 1980, which was the first model to implement a ‘shift invariant’ algorithm, i.e. detection of features was unaffected by the location of the feature in the image^16^. The name ‘convolutional neural network’ (CNN) was proposed by Lecun et al., who used the convolution operation and constructed a simple network (LeNet) consisting of convolution-pooling-convolution-pooling-dense-dense layers, for handwritten digit recognition tasks^17^. However, image classification gained traction since publication of the ImageNet database, a large, multi-class collection of images, in 2009. The first influential CNN model, AlexNet, an 8-layered CNN, achieved 15.3% error-rate on the ImageNet database ^18^. After a few years, The Visual Geometry Group (VGG)^19^proposed several deep neural networks (11 to 19 layers deep) which achieved 7.3% error rate on the ImageNet database. In 2014, the Inception block architecture (a local unit with parallel branches of convolution) was introduced by Google Inc. in their models Inception V1 to V4, which reduced the error rate to 6.7%^20^. A slight improvement on the Inception model was proposed by Chollet in 2017, introducing depthwise convolution followed by pointwise convolution, termed as the Xception model^21^. The Xception model achieved 79% top-1 accuracy on the ImageNet database, and has been employed in several other problem domains such as finger vein recognition^22^ and plant disease classification^23^. The CNN paradigm was further improvised with the introduction of residual nets (skip connection between layers) in 2015 by He et al.^24^ In a further improvement, Tan et al., in 2019, developed the EfficientNet, which implemented a new scaling method for CNNs, achieving 88% top-1 accuracy in Imagenet^25^. A variant, the FixEfficientNet by Touvron et al., which corrects discrepancy between training and test images, achieved 98.7% top-5 accuracy on ImageNetV2^26^. In early 2020s, there was another paradigm shift in image classification: Dosovitskiy et al. proposed implementing the transformer architecture, used in natural language processing tasks, in image classification^27^. Such Vision Transformers (ViTs) have now become state of the art in image classification, producing 90.94% top-1 accuracy on ImageNet database^28^.

Similar developments have taken place in feature extraction and enhancement of images. The extreme gradient boosting classifier (XGBoost), developed by Chen et al.^29^, was used on the Caltech-101 image dataset producing high accuracy (88.36%), outperforming most other major feature detectors^30^. Kumar et al. have demonstrated feature extraction from images has advanced sufficiently to retrieve images from large datasets based on content, with 99.53% precision^31^. Low contrast images, such as underwater photographs, have been demonstrated to be enhanced by the Contrast-Limited Adaptive Histogram Equalization (CLAHE) algorithm, which is a significant improvement over conventional histogram equalisation method^32,33^. Alternative approaches to image classification, such as the Shi-Tomasi corner detection algorithm^34^ along with the scale-invariant feature transform (SIFT)^35^, have produced 86.4% accuracy on the Caltech-101 image dataset^36^. It’s also been demonstrated that image analysis techniques can detect the device of origin (i.e. camera brand) with reasonable accuracy^37^. Such advances in image analysis techniques have complemented the ML models for successful image classification.

Neural networks are not limited to the domain of image analysis; they have also found application in signal processing, such as biometric identification through electrocardiogram (ECG)^38,39^ and analysis of electroencephalograms^40^, often in conjunction with other ML models such as support vector machines and k-nearest neighbours^41^. The generic nature of neural networks provides them with extensibility to suit diverse problem domains.

In the specific field of histology, the application of deep neural networks has been explored in recent studies, albeit in a fragmentary manner. A limited histology classifier was developed by Rujano-Balza et al., which classified into five basic types (epithelium, gland, connective, muscular and nervous tissue)^42^. The VGG16 model, the one we have used in the present study, has been previously used in classifying breast cancer histology images with 92.60% accuracy (albeit as a binary classifier: benign versus malignant)^43^. Ahmed et al. have used the VGG16 and InceptionV3 pretrained models to classify histological images from a mixed dataset (Kimia Path 24)^44^; however, their method was not targeted to identify specific tissues (the Kimia Path dataset is a mixed set of images from different tissues with different stains). We identified that there was a definite lacuna in literature regarding comparison of human versus ML performance in this specific domain. Thus we took the approach of multi-class tissue classification on a defined set of tissues, similar to the training imparted to medical students in first year of medical school.

### Problem statement

We wanted to compare the performance of two kinds of ‘agents’ on a novel dataset. The agents are.

1. Machine learning model (ML)—two pretrained CNNs.

2. A group of medical students (MS).

**Figure 1:**
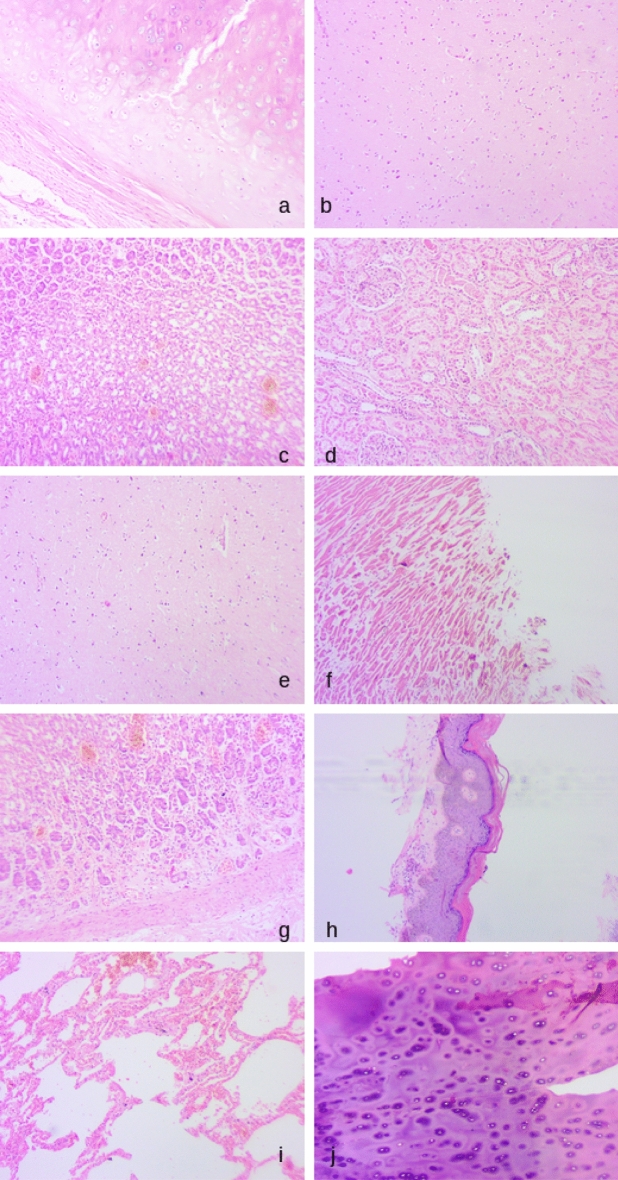
Random sample of images used in the study: (**a**). Trachea, (**b**). Cerebrum, (**c**). Stomach, (**d**). Kidney, (**e**). Cerebrum, (**f**). Heart, (**g**). Stomach, (**h**). Skin, (**i**). Lungs, (**j**). Trachea (Composite figure generated with ImageMagick, version 7.1.0, https://imagemagick.org/index.php).

We have compared the performance of MS and ML models on a *validation* set – composed of tissues within the scope of training; we have also compared their performance on a smaller set of images *outside of the scope of training*, i.e. a completely different set of tissues not included in training. A random sample of images used in the study (and included in the training set) is shown in Fig. 1.

The objective of the present study was to (a) compare the accuracy of ML models and medical students in an image classification problem, on a dataset which is novel to both humans and ML models, (b) compare the pattern of errors of ML models and medical students and thus find similarities, if any. This would provide an insight whether there are similarities between the inner representation of visual information in humans and ML models.

## Materials and methods

### Ethics statement

Ethical clearance was obtained from Institutional Ethical Committee, Base Hospital and Army College of Medical Sciences, Delhi Cantonment, Delhi, India (No. IEC/01/2021/10). The study involved voluntary and anonymous participation by medical students in an online quiz; students were asked by the faculty of Dept of Pathology, Army College of Medical Sciences, to participate in the study on a purely voluntary basis. **Informed consent was obtained from participants via electronic medium**. The quiz was fully anonymised: no personal information, which could potentially reveal the identity of the candidates, was collected in the online quiz.

The study did not involve any kind of diagnostic/therapeutic modality on human subjects. A group of adult medical students were asked to voluntarily and anonymously participate in an online quiz, hosted by Army College of Medical Sciences, after adequate information to students. **All relevant guidelines regarding participation of human subjects, including the Helsinki declaration, were followed.**

### Preparation of image dataset

Histological images from tissues were collected from the archives of a hospital in North India. An Olympus Magcam Microphotography system was used for acquiring the images. All images were acquired with a Dewinter 606 Trinocular Microscope, under the same condition of illumination and 10 × maginification. The images were acquired in 1280 × 960 pixels resolution and resized to 512 × 384 pixels with ImageMagick image processing software^45^. All histological slides were anonymised before image acquisition.

**Figure 2:**
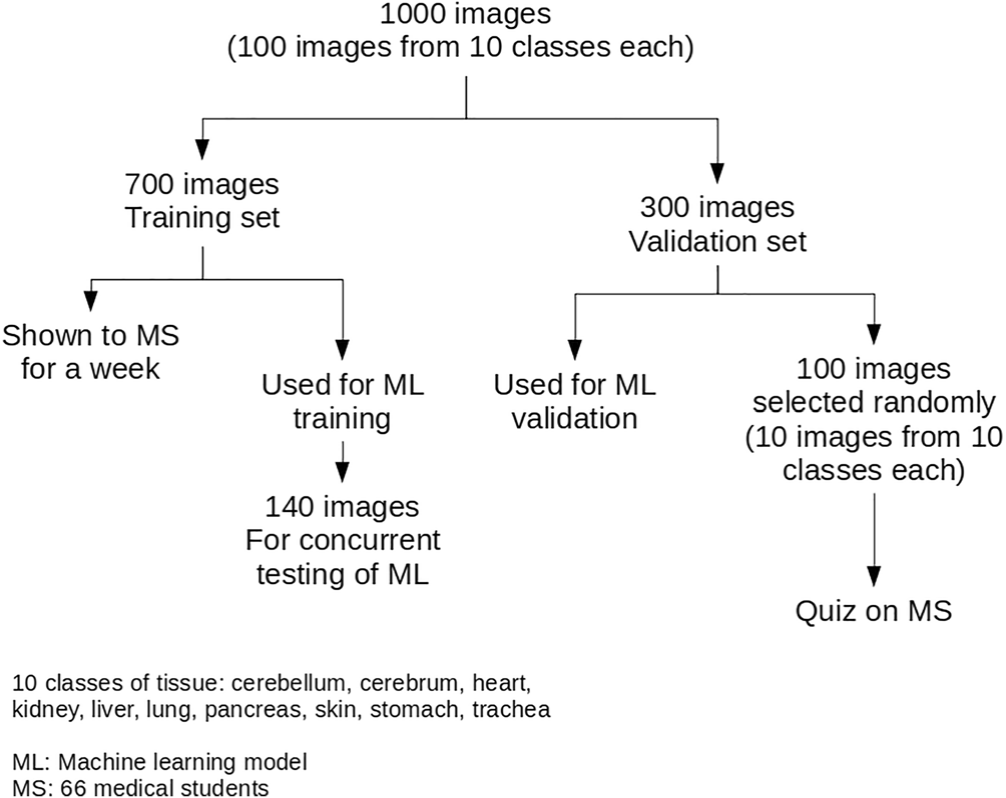
Schematic diagram of the study (Figure generated with Libreoffice Draw, version 7.2.7, https://www.libreoffice.org/).

For selection of tissues for inclusion in the study, we made a list of tissues which are introduced to the medical students in first year curriculum, and randomly selected 10 classes from them. Images from the following ten classes of tissue were acquired: cerebellum, cerebrum , heart , kidney , liver , lung , pancreas , skin , stomach , trachea. One hundred (100) images from each class were acquired, for a total of one thousand (1000) images. The schematic diagram of the study is presented in Fig. 2.

The image dataset was randomly split into two subsets (using the ‘shuf’ program of the Linux operating system)^46^:

I. A *training* set of 700 (seven hundred) images, consisting of 70 images from each class of tissue.

- Within the training set, 20% images (140 images) were selected randomly (using the *train_test_split* function from the *scikit-learn* Python library, version 1.0.2^47^) as *test* set, for concurrent testing during training of ML model.

II. A *validation* set of 300 (three hundred) images, consisting of 30 images from each class of tissue.

- From the validation set, 100 images were marked for inclusion in the *quiz* set, i.e. for testing human participants; the quiz set contained 10 images randomly selected from each of the classes ‘cerebellum', ‘pancreas', ‘kidney', ‘trachea', ‘cerebrum', ‘lung', ‘heart', ‘skin', ‘liver' and ‘stomach'.

### Construction of ML model

Two ML models were constructed using the DNN architecture. We used transfer learning in constructing the ML model, i.e. used the convolutional layers of a pretrained model for image analysis (i.e. to extract features of the image). Initially, we shortlisted two models, pre-trained VGG16 and ResNet50, because both of them are extensively trained with ImageNet database and frequently used in image recognition tasks. The output of the convolution-pooling layers from both models was flattened into a one dimensional array, and several fully connected and dropout layers were added to produce a final output layer of 10 classes. During initial testing, the modified ResNet50 model did not exceed 50% accuracy on the concurrent testing set, and thus it was not selected for the study. We selected VGG16 as our model, because in addition to its good performance on the testing set, it has been used previously in histological classification problems (Shallu^43^). In addition, VGG16 was used by a similar study by Dodge et al., which used everyday images (i.e. dog breeds) and their distorted versions, for human–machine comparison^12^.The architecture of the model is shown in Table 1. In joining the convolution – pool layers of VGG16 and the fully connected layers, the method published by Rosebrock^48^ and Brownlee^49^ was followed.

**Table 1:** Architecture of the VGG16 model; Total parameters: 18,128,714, trainable parameters: 3,414,026 ; non-trainable parameters: 14,714,688; the junction between the convolution-max pooling layers of the original VGG16 model and the fully connected layers attached to its output – is shown in bold. The final output layer produces 10 numbers, corresponding to the probabilities of a given image belonging to the 10 classes of tissue.

Layer (type)	Output Shape	Parameters
input_3 (InputLayer)	[(None, 128, 96, 3)]	0
block1_conv1 (Conv2D)	(None, 128, 96, 64)	1792
block1_conv2 (Conv2D)	(None, 128, 96, 64)	36928
block1_pool (MaxPooling2D)	(None, 64, 48, 64)	0
block2_conv1 (Conv2D)	(None, 64, 48, 128)	73856
block2_conv2 (Conv2D)	(None, 64, 48, 128)	147584
block2_pool (MaxPooling2D)	(None, 32, 24, 128)	0
block3_conv1 (Conv2D)	(None, 32, 24, 256)	295168
block3_conv2 (Conv2D)	(None, 32, 24, 256)	590080
block3_conv3 (Conv2D)	(None, 32, 24, 256)	590080
block3_pool (MaxPooling2D)	(None, 16, 12, 256)	0
block4_conv1 (Conv2D)	(None, 16, 12, 512)	1180160
block4_conv2 (Conv2D)	(None, 16, 12, 512)	2359808
block4_conv3 (Conv2D)	(None, 16, 12, 512)	2359808
block4_pool (MaxPooling2D)	(None, 8, 6, 512)	0
block5_conv1 (Conv2D)	(None, 8, 6, 512)	2359808
block5_conv2 (Conv2D)	(None, 8, 6, 512)	2359808
block5_conv3 (Conv2D)	(None, 8, 6, 512)	2359808
block5_pool (MaxPooling2D)	(None, 4, 3, 512)	0
**flatten_2 (Flatten)**	**(None, 6144)**	**0 **
dense_4 (Dense)	(None, 512)	3146240
dropout_4 (Dropout)	(None, 512)	0
dense_5 (Dense)	(None, 512)	262656
dropout_5 (Dropout)	(None, 512)	0
class_label (Dense)	(None, 10)	5130

**Figure 3:**
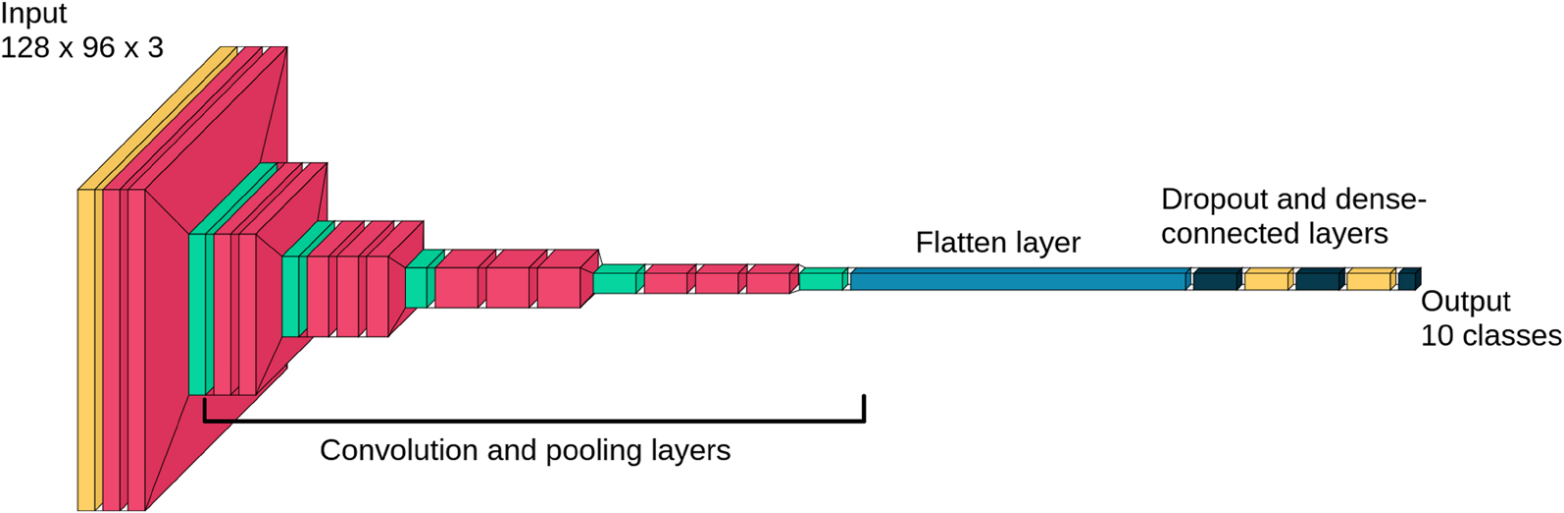
Graphical representation of the modified VGG16 model; the model takes a 128 × 96 color image as input (extreme left) and converts it to a 10 class output (extreme right); the dark blue block represents the flattened output of the convolution and max pooling layers of VGG16, which is followed by fully connected and dropout layers (Figure produced with the Visualkeras python library, version 0.0.2, https://github.com/paulgavrikov/visualkeras).

The input image was preprocessed with the OpenCV image processing software^50^, which consisted of resizing the color image to 128 × 96 pixels. The DNN model takes this numeric array as input; thus its input dimension is 128 × 96 × 3 (the third dimension corresponds to the three color channels, red, green and blue, at each pixel). After several convolution and max-pooling layers, the array is reshaped to a shape of 4 × 3 × 512. This layer is flattened to a one-dimensional array of size 6144. Several fully connected and dropout layers then reduce the array to a final size of 10. Figure 3 shows the architecture of the modified VGG16 model, represented graphically.

The model was constructed with the Keras deep learning library (version 2.8.0)^51^ in Python programming language, using the Google Colaboratory platform.

In addition, after rejection of ResNet model, we used another pretrained model (Inception V2) from the Keras applications library; we modified it in a similar manner as the VGG16 model, i.e. flattened its output layer and added a series of dense and dropout layers. The structure of the modified inception model is shown in Table 2.

**Table 2:** The InceptionV2 model with 55,330,922 trainable parameters; the weights from ImageNet training have been preserved and a series of dense and dropout layers added to match the present 10-class classification problem.

Layer (type)	Output shape	Number of parameters
inception_resnet_v2 (Functional)	(None, 1536)	54,336,736
flatten_6 (Flatten)	(None, 1536)	0
dense_12 (Dense)	(None, 512)	786,944
dropout_8 (Dropout)	(None, 512)	0
dense_13 (Dense)	(None, 512)	262,656
dropout_9 (Dropout)	(None, 512)	0
class_label (Dense)	(None, 10)	5130

### Training of VGG 16 model

**Figure 4:**
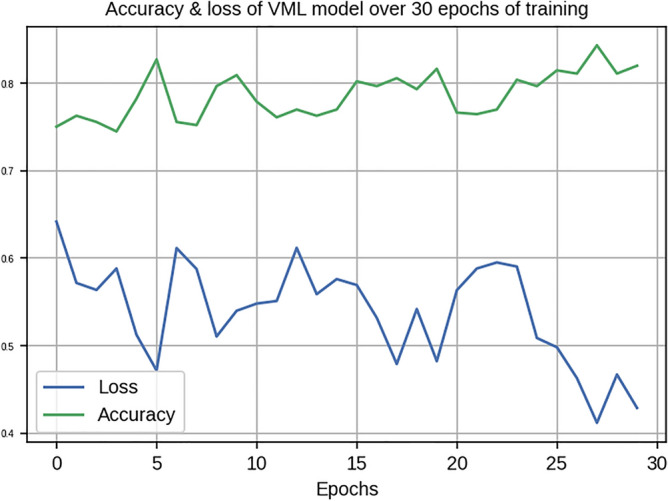
Accuracy and loss (error rate) of the VML over 30 epochs of training (Figure generated by Matplotlib python library, version 3.3.4, https://matplotlib.org/).

The model thus developed was trained with the training dataset over 30 epochs, with a batch size of 10 images. At the end of training, the model achieved 81.96% accuracy over the training test, and 77.14% accuracy in the concurrent test set. Accuracy and loss (error rate) of the model over epochs of training are shown in Fig. 4.

### Evaluation of VGG16 ML (VML) model

The VGG16 model (VML) was evaluated on the validation set (*n* = 300), which yielded 85.67% accuracy. The confusion matrix on the validation set is shown in Table 3:

**Table 3:** Confusion matrix or actual and predicted labels (VML model on validation set, *n* = 300).

Predicted	Cere-bellum	Cerebrum	Heart	Kidney	Liver	Lung	Pancreas	Skin	Stomach	Trachea	Total (actual)
Actual											
Cere-bellum	29	0	0	0	0	0	1	0	0	0	30
Cerebrum	2	28	0	0	0	0	0	0	0	0	30
Heart	0	0	25	0	1	1	2	0	1	0	30
Kidney	0	0	0	22	8	0	0	0	0	0	30
Liver	0	0	0	2	28	0	0	0	0	0	30
Lung	0	0	0	0	0	30	0	0	0	0	30
Pancreas	2	0	0	0	3	0	25	0	0	0	30
Skin	0	0	0	0	0	0	0	29	1	0	30
Stomach	0	0	4	1	8	0	2	0	15	0	30
Trachea	1	0	0	0	0	1	0	1	1	26	30
Total (predicted)	34	28	29	25	48	32	30	30	18	26	300

The VGG16 model (VML) classified 257 of the 300 images in the validation set correctly (85.67%). The commonest error by the VGG16 model was (a) misclassifying stomach as liver (08 images) and (b) misclassifying kidney as liver (08 cases) and (c) misclassifying stomach as heart (04). The best accuracy was recorded in recognising lung (30/30, 100%) and the worst in stomach (15/30, 50%).

**Figure 5:**
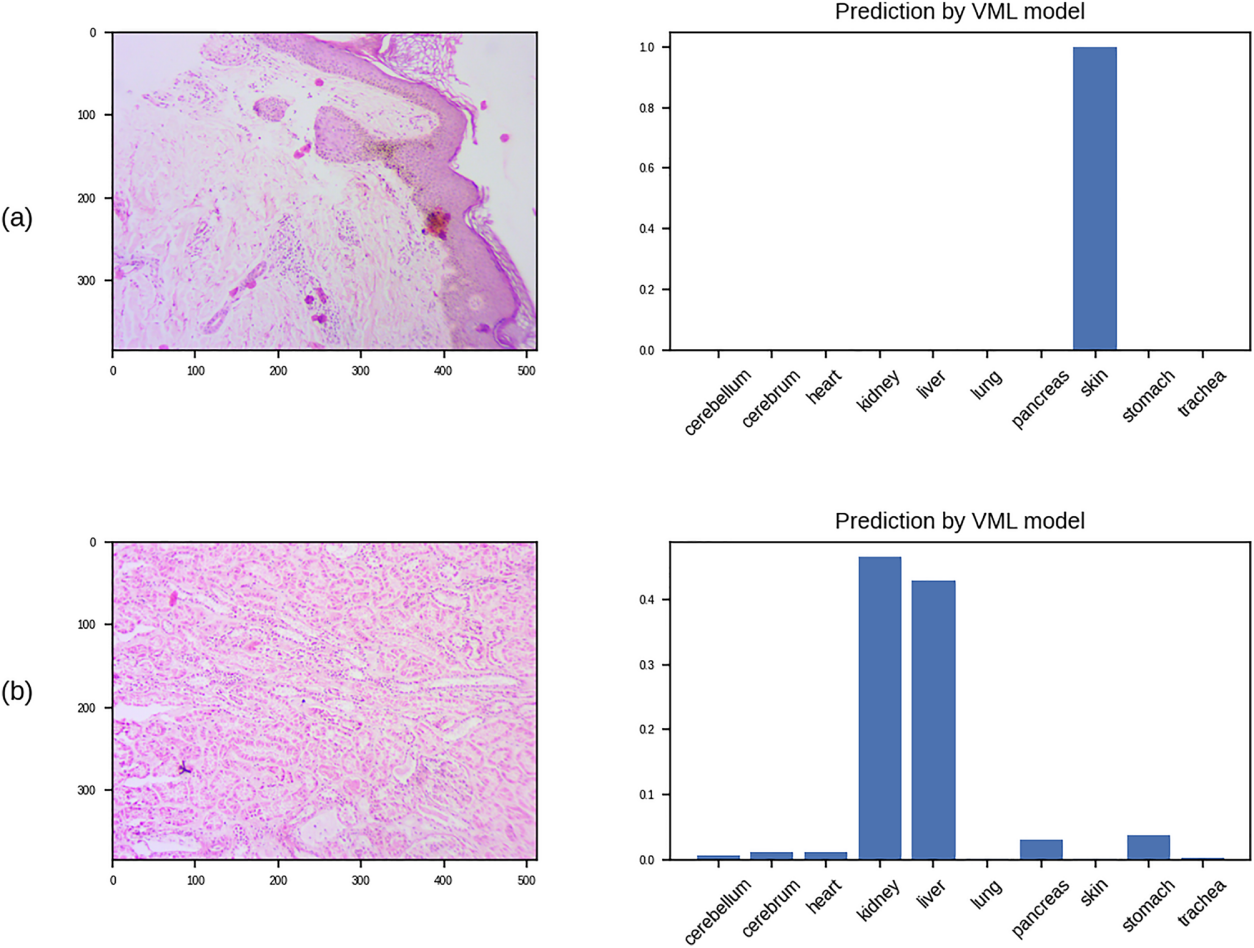
Examples of classification by VML: (**a**) Skin, correctly identified by the VML; the artifact in the section has not interfered with correct classification by the VML; (**b**) Kidney tissue as classified by the VML; the model generates 10 numbers as output, corresponding to the probability of 10 classes of tissue. In this case, 'liver' is a close second (Figure generated by Matplotlib python library, version 3.3.4, https://matplotlib.org/).

Figure 5 shows examples of correct classification by the VML. The VML outputs 10 numbers, corresponding to the probabilities of the image belonging to the respective class of tissue. In Fig. 5a, the classification is unequivocal, i.e. there is a very high probability (close to 1) of the tissue being ‘skin’. In Fig. 5b, the VML has generated various probabilities for different classes of tissue, i.e. the output is equivocal, and the class with the highest probability (‘kidney’) has been chosen as the correct answer.

### Training of inception V2 model (IML)

**Figure 6:**
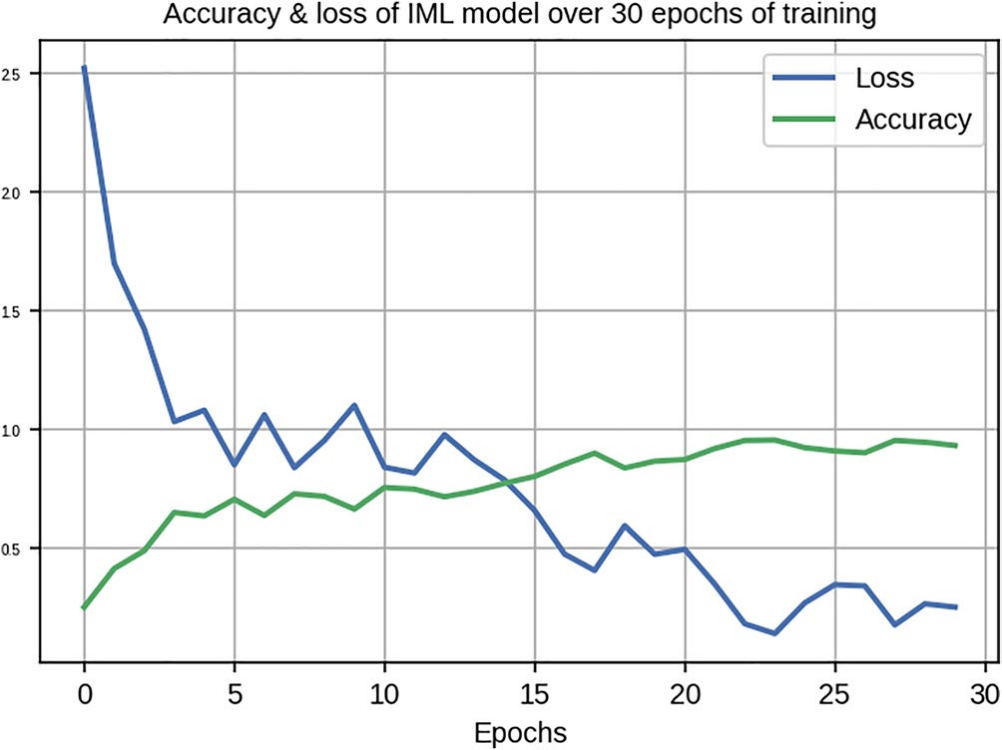
Accuracy and loss over 30 epochs of training the Inception model; this model showed a much sharper drop in error rate (loss) during the early epochs, than the VML model (Fig. 4) (Figure generated by Matplotlib python library, version 3.3.4, https://matplotlib.org/).

The inception V2 model was trained with the same training dataset as the VGG16 model with same hyperparameters (30 epochs, with a batch size of 10 images). The model achieved 89% accuracy on the concurrent testing set (Fig. 6).

### Evaluation of Inception model

The Inception model (IML) achieved 90.33% (271 out of 300) accuracy on the validation set. In contrast to the VGG16 model, the Inception model made maximum number of errors in recognising the ‘skin’ class (8 errors out of 29, 27.5%), followed by ‘stomach’ class (7 errors). (Table 4) Examples of classification by the IML model are shown in Figs. 19, 21 and 23.

**Table 4:** Confusion matrix of IML on the validation set (*n* = 300).

Predicted	Cere-bellum	Cerebrum	Heart	Kidney	Liver	Lung	Pancreas	Skin	Stomach	Trachea
Actual										
Cere-bellum	30	0	0	0	0	0	0	0	0	0
Cerebrum	0	30	0	0	0	0	0	0	0	0
Heart	0	0	26	0	0	0	0	0	4	0
Kidney	0	0	0	30	0	0	0	0	0	0
Liver	0	0	0	5	25	0	0	0	0	0
Lung	0	0	0	0	0	30	0	0	0	0
Pancreas	0	0	0	0	0	0	30	0	0	0
Skin	0	0	0	7	0	0	0	22	1	0
Stomach	0	0	0	4	3	0	0	0	23	0
Trachea	0	0	1	0	0	1	0	2	1	25

### Training of medical students (MS)

The medical students (MS) of who have just completed first year in a Medical School in North India were asked to volunteer for the study. We circulated the Participation Information Sheet by electronic messaging through the class representative. It was explained to the students that the very act of submitting the electronic questionnaire will be taken as consent for participation in the study. The purpose and objective of the study was explained to the class, and that anonymised data from the study will be published. Informed consent was obtained from participants via electronic medium**.** The students were trained with basic histological tissue identification for a period of 02 months before the study, as part of their curriculum in 1^st^ year MBBS course. The training of medical students in histology was imparted in a heuristic manner as is conventional in histology: specific identification criteria were taught to the students—i.e. ‘liver’ was to be identified by its lobular structure and kidney by glomeruli etc. In addition, the complete set of 700 training images were circulated to medical students one week prior to the quiz.

In our present medical curriculum, first year medical students are introduced to human histology. Majority of these students have never seen histological images of human tissues before, and are thus a naive population. Their performance in recognising histological images is very similar to a naive, untrained ML model. These students are ideal candidates for training with a set of histological images and assess the resulting efficacy, for direct comparison with a similarly trained ML model. By the time they complete second year of medical school, they have already been well trained with histological images as part of the pathology curriculum, and thus cannot be compared with an ML model.

### Preparation of questionnaire

The ‘quiz’ set (IIa) of images (100 images) were incorporated into an online quiz format (built with Microsoft Office Forms); a set of 100 questions were formulated, with a choice of 10 options each, corresponding to the ten classes of tissue. Answering all questions was not mandatory (and all such ‘blank’ or ‘other’ answers were excluded from final analysis); no time limit was set on the questionnaire. (Link to the questionnaire is provided in the ‘Data availability statement’ section).

### Conduct of survey

The quiz questionnaire was shared online with medical students. The online survey was conducted throughout a week. Strict anonymisation was maintained in data collection; no personal information was collected from the survey. However, students were strictly instructed to respond to the quiz only once per person, i.e. no duplication in responses was allowed. At the end of the week, sixty-six (66) responses were aggregated and tabulated.

### Preparation of out-of-training-scope (OTS) image set

As adversarial examples, meant to test the pattern recognition capabilities of ML and MS, 10 images from tissues *which were not part of the training data* were separately selected from archives of the hospital. The 10 images were as follows (Table 5):

**Table 5:** Out-of-training-scope (OTS) images.

1	Parathyroid
2	Cervix
3	Intestine
4	Intestine
5	Parathyroid
6	Intestine
7	Salivary gland
8	Endometrium
9	Salivary gland
10	Cervix

The OTS images were selected to highlight the differences between ML and MS. The ‘intestine’ class was included because of its similarity to the ‘stomach’ class, and also that medical students are likely to have seen images of intestine before, while the ML model has never been trained with ‘intestine’ class. The ‘cervix’ class was chosen because of its similarity with ‘skin’, and ‘salivary gland’ because of its similarity to ‘pancreas’. The parathyroid and endometrium was unlike any tissue the students or ML models would have encountered during training.

### Evaluation of ML and MS on OTS images

Both ML models were run on the OTS images and the predictions were recorded. A second online quiz was conducted on the medical students with the OTS image set. 44 responses were recorded.

### Informed consent

The study involved **voluntary and anonymous participation** by medical students in an online quiz; students were asked by the faculty of Dept of Pathology, Army College of Medical Sciences, to participate in the study on a purely voluntary basis. **Participant information sheet (PIS)** was circulated to the students; **informed consent was obtained from participants via electronic medium**. We circulated the Participation Information Sheet by electronic messaging through the class representative. **It was explained to the students that the very act of submitting the electronic questionnaire will be taken as consent for participation in the study**. The purpose and objective of the study was explained to the class, and that anonymised data from the study will be published.

## Results

### Performance of VML model on quiz questionnaire

The VGG16 ML model achieved 91% accuracy in the quiz (Table 6). The most common error encountered was misclassifying kidney tissue as liver. The model showed poorest performance in recognising stomach: misclassifying stomach tissue as heart (02 images), liver (01 image) and pancreas (01 image).

**Table 6:** Confusion matrix of the predictions of VGG16 ML model on quiz questionnaire (n = 100).

Predicted	Cere-bellum	Cerebrum	Heart	Kidney	Liver	Lung	Pancreas	Skin	Stomach	Trachea	Total (actual)
**Actual**											
Cere-bellum	10	0	0	0	0	0	0	0	0	0	10
Cerebrum	0	10	0	0	0	0	0	0	0	0	10
Heart	0	0	10	0	0	0	0	0	0	0	10
Kidney	0	0	0	7	3	0	0	0	0	0	10
Liver	0	0	0	0	10	0	0	0	0	0	10
Lung	0	0	0	0	0	10	0	0	0	0	10
Pancreas	0	0	0	0	0	0	10	0	0	0	10
Skin	0	0	0	0	0	0	0	9	1	0	10
Stomach	0	0	2	0	1	0	1	0	6	0	10
Trachea	1	0	0	0	0	0	0	0	0	9	10
Total (predicted)	11	10	12	7	14	10	11	9	7	9	100

### Performance of IML model on quiz questionnaire

The IML model produced 93% accuracy on the quiz questionnaire (Table 7). In contrast to the VML model, the commonest error of the IML model is recognising ‘skin’ (03 errors), which was variably mistaken as ‘kidney’ (02 errors) or ‘stomach’ (01 error).

**Table 7:** Confusion matrix of the IML model on the quiz questionnaire (n = 100); as in the validation set, the commonest errors were met in recognising skin (03 errors).

Predicted	cerebellum	cerebrum	Heart	Kidney	Liver	Lung	Pancreas	Skin	Stomach	Trachea	Total (actual)
**Actual**											
cerebellum	10	0	0	0	0	0	0	0	0	0	10
Cerebrum	0	10	0	0	0	0	0	0	0	0	10
Heart	0	0	10	0	0	0	0	0	0	0	10
Kidney	0	0	0	10	0	0	0	0	0	0	10
Liver	0	0	0	2	8	0	0	0	0	0	10
Lung	0	0	0	0	0	10	0	0	0	0	10
Pancreas	0	0	0	0	0	0	10	0	0	0	10
Skin	0	0	0	2	0	0	0	7	1	0	10
Stomach	0	0	0	1	0	0	0	0	9	0	10
Trachea	0	0	0	0	0	1	0	0	0	9	10

### Performance of medical students on quiz questionnaire

The combined performance of 66 medical students on the quiz questionnaire (a total of 6557 responses, excluding blank / ‘other’ answers) is shown in Table 8. The overall accuracy of the medical students (MS) was 55.14%.

**Table 8:** Confusion matrix of the aggregated responses from 66 medical students (MS) over quiz questionnaire (*n* = 6557).

Answered	Cere-bellum	cerebrum	Heart	Kidney	Liver	Lung	Pancreas	Skin	Stomach	Trachea	Total (actual)
**Actual**											
Cerebellum	336	101	32	25	22	28	39	29	23	19	654
Cerebrum	49	406	31	23	35	28	28	27	23	9	659
Heart	23	26	386	36	33	29	32	35	40	14	654
Kidney	20	17	28	441	19	26	52	18	25	10	656
Liver	14	20	34	61	301	33	112	31	36	14	656
Lung	21	21	28	29	24	433	42	20	22	13	653
Pancreas	19	42	40	65	58	28	316	25	46	15	654
Skin	42	34	33	23	17	29	22	340	36	80	656
Stomach	16	24	33	59	61	28	160	18	241	18	658
Trachea	21	33	22	36	15	27	31	36	20	416	657
Total (answered)	561	724	667	798	585	689	834	579	512	608	6557

**Figure 7:**
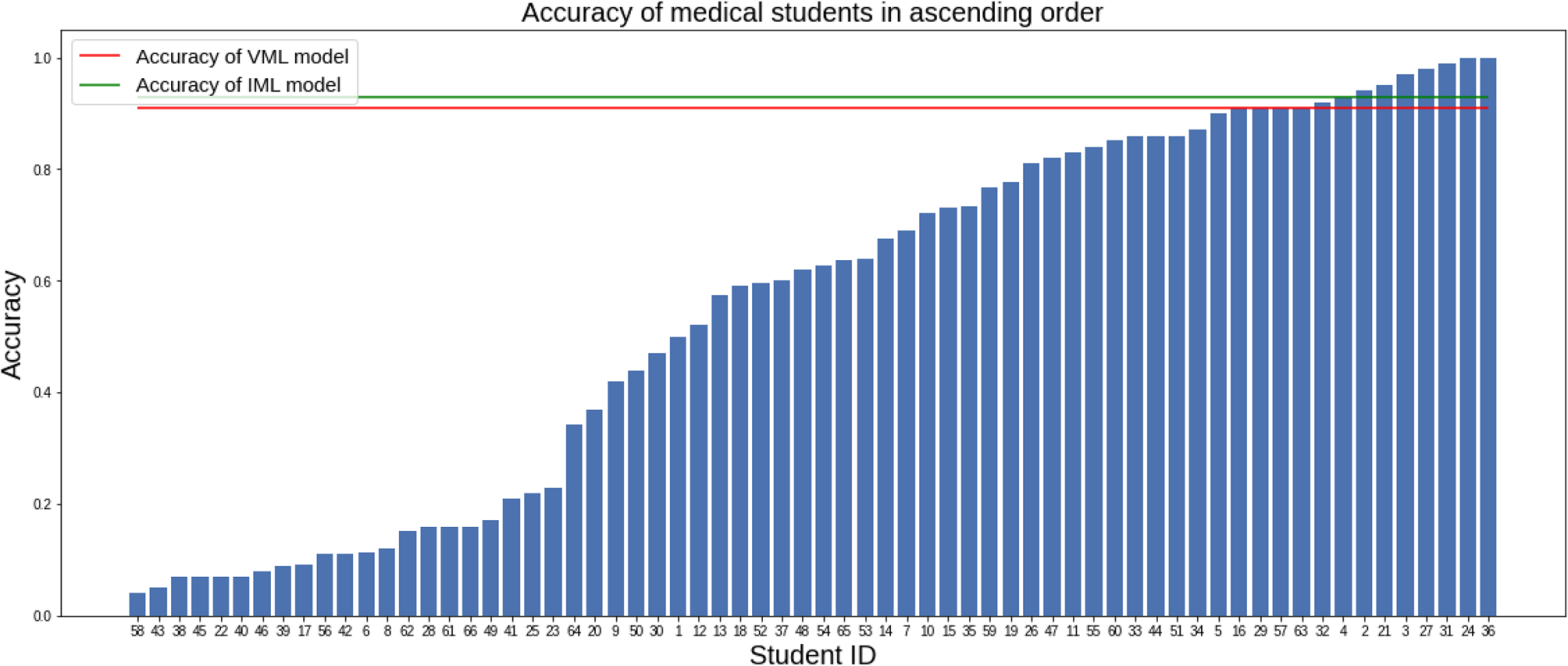
Accuracy of 66 medical students in ascending order (Figure generated by Matplotlib python library, version 3.3.4, https://matplotlib.org/).

Analysing by responses from individual students, accuracy of students ranged from 4 to 100%. 13 medical students out of 66 (19.67%) matched or exceeded the accuracy of the VML model, i.e. the VML model was at 80th percentile if compared with medical students (Fig. 7). Only 08 medical students (12.12%) scored better than the IML model.

**Figure 8:**
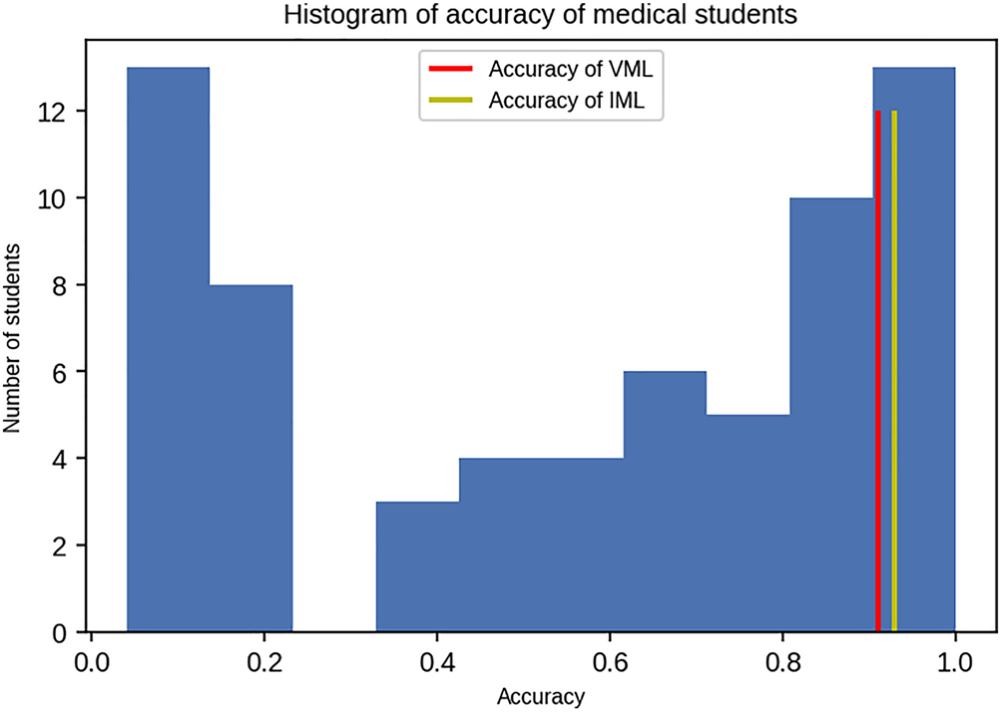
Histogram of accuracy of 66 medical students in the quiz questionnaire; the accuracy of VML and IML are shown for comparison (Figure generated by Matplotlib python library, version 3.3.4, https://matplotlib.org/).

A histogram of the accuracy of medical students shows concentration of scores at two extremes of accuracy (Fig. 8).

### Comparison of ML models and medical students on questionnaire

**Figure 9:**
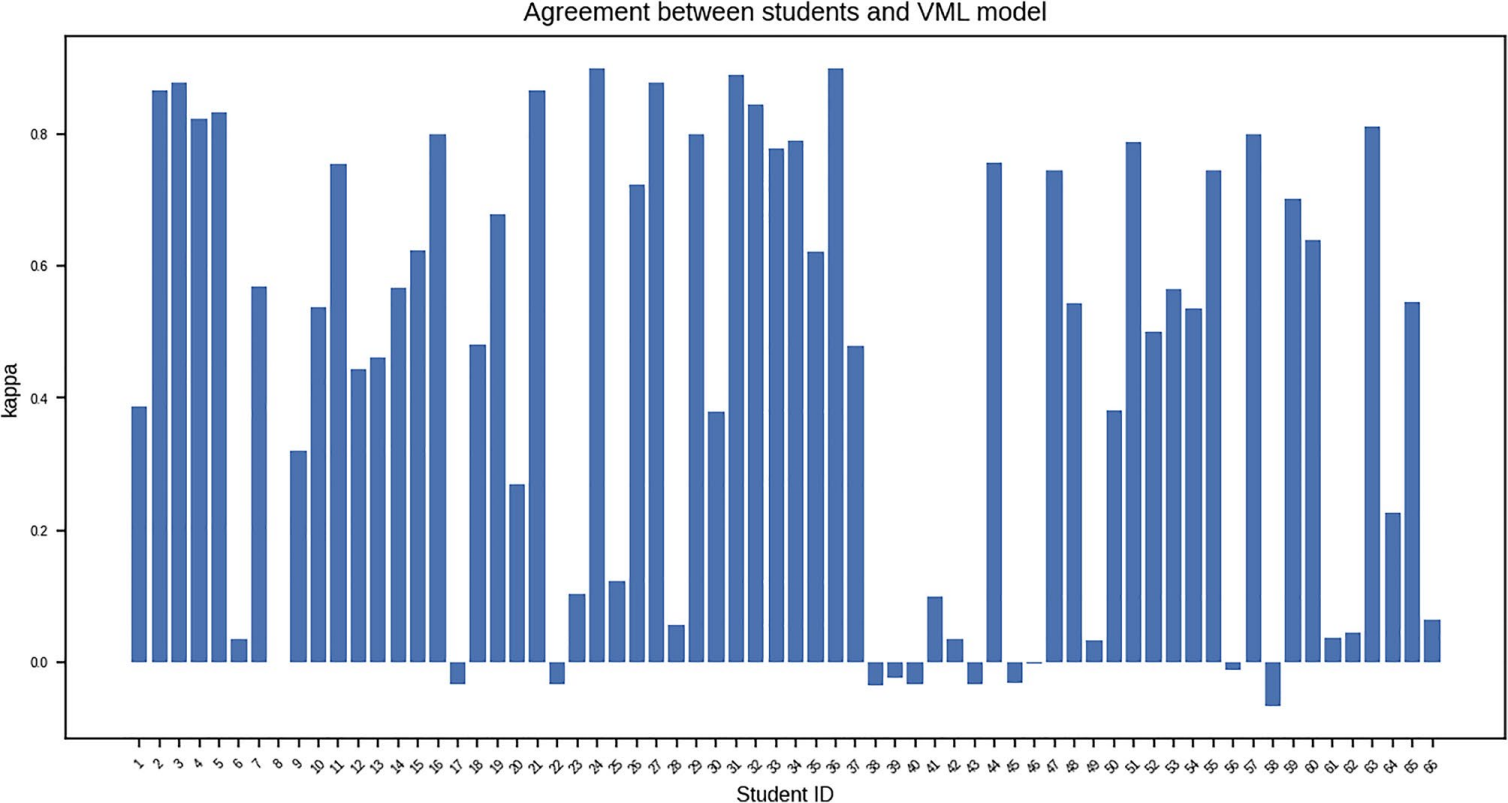
Plot of Cohen’s kappa for each student (n = 66), as a measure of agreement between students and VML model (Figure generated by Matplotlib python library, version 3.3.4, https://matplotlib.org/).

Analysis of responses, student-by-student, shows variable agreement with the VML model (Cohen’s kappa ranging from − 0.06 to + 0.9). Negative kappa values were seen in Participants 58, 38, 39, 40, 43, 45 – i.e. the same students who had achieved lowest test scores (Fig. 9).

A tissue-wise comparison between MS (aggregate of all responses) and both ML models (VML & IML) showed the classification by ML models to be significantly different from MS; results of tissue-wise Chi-square Goodness of Fit are shown (Table 9):

**Table 9:** Tissue wise agreement (Chi squared Goodness of Fit) between MS, VML & IML in the quiz questionnaire (*n* = 100).

Tissue class	Comparison between MS and VML		Comparison between MS & IML	
	Chi square	*p*-value	Chi square	*p*-value
Cerebellum	539.34	2.14e-110 *	541.297619	8.16e-111*
Cerebrum	704.25	8.45e-146 *	704.246305	8.45e-146*
Heart	643.38	1.01e-132 *	647.259067	1.49e-133*
Kidney	784.11	5.59e-163 *	768.483193	1.28e-159*
Liver	557.82	2.34e-114 *	569.212625	8.44e-117*
Lung	669.23	2.83e-138 *	667.267984	7.49e-138*
Pancreas	812.32	4.73e-169 *	814.316456	1.76e-169*
Skin	561.24	4.33e-115 *	565.144118	6.29e-116*
Stomach	498.18	1.41e-101 *	492.363877	2.48e-100*
Trachea	590.19	2.66e-121 *	590.194712	2.66e-121*

Significance of *: indicates statistically significant values.

#### Image-by-image comparison of equivocal responses by MS & VML

**Figure 10:**
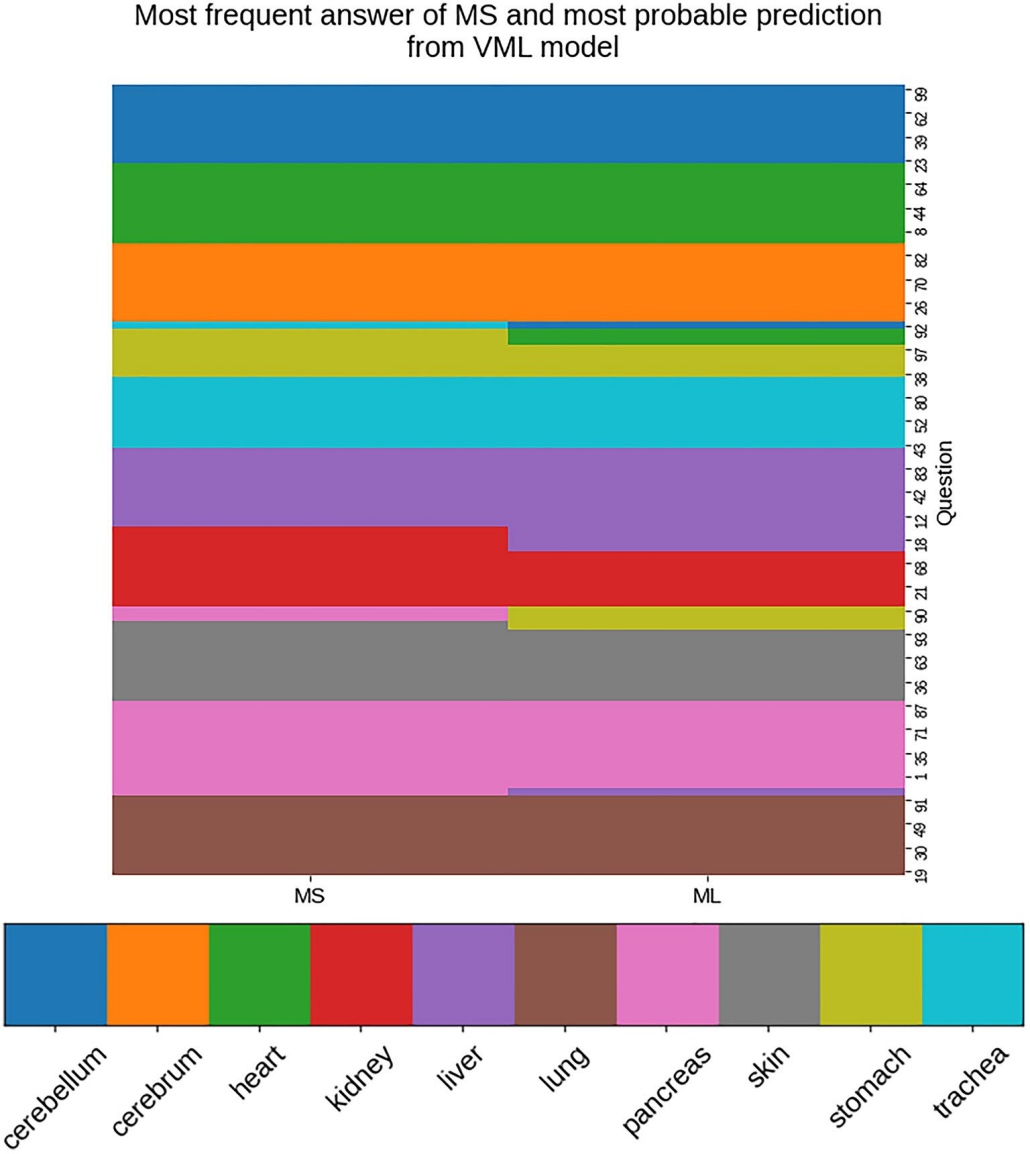
Clustered heatmap showing commonest responses by MS, and predictions with highest probability from VML, on each individual image of the quiz set (n = 100); 90% match is noted (Figure generated by Matplotlib python library, version 3.3.4, https://matplotlib.org/).

Figure 10 shows the most frequent answer among all medical students, and the most probable prediction from VML model, for each image, as a clustered heatmap.

**Figure 11:**
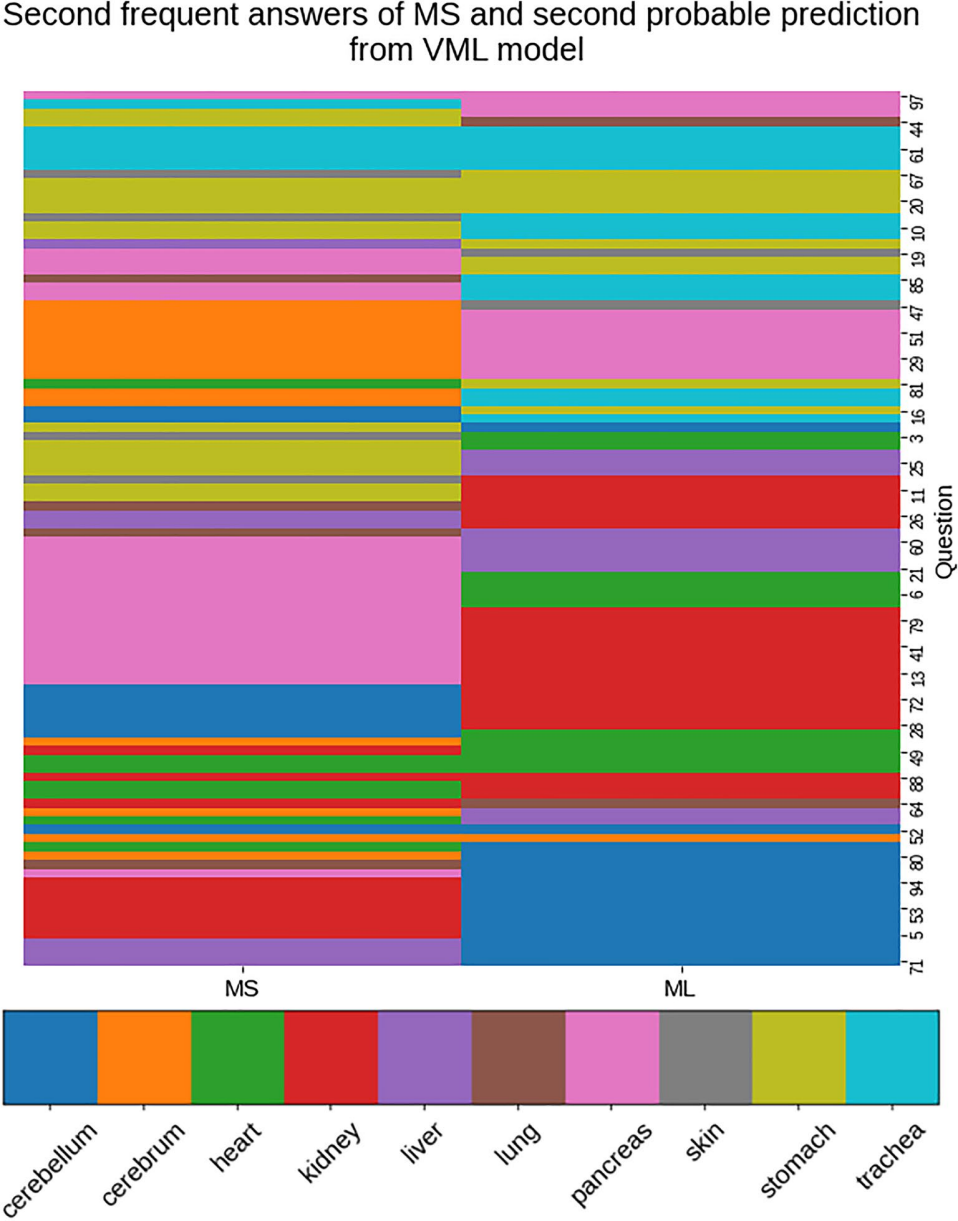
The second commonest answer of the medical students, and the prediction from VML model with second highest probability—matched only in 15 images of the quiz questionnaire (*n* = 100); in 14 of these images, the first prediction also matched (Figure generated by Matplotlib python library, version 3.3.4, https://matplotlib.org/).

The comparison between 2nd commonest answers from MS, and predictions with 2^nd^ highest probability from VML, image-by-image, is shown in Fig. 11.

**Figure 12:**
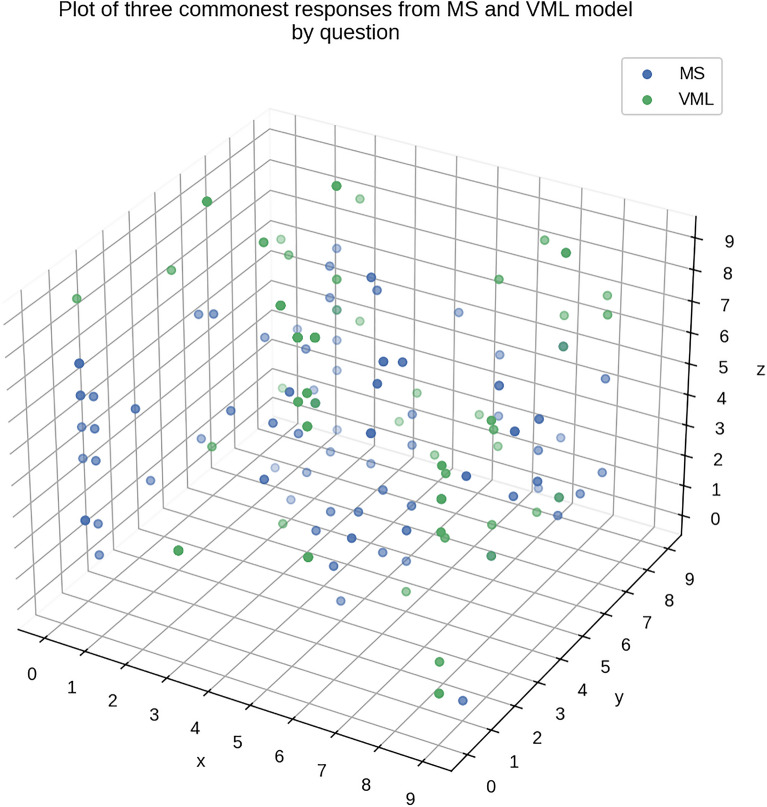
Plot of three commonest responses from MS and VML. Each dot represents a question from the quiz. The x axis represents the commonest response, y axis – the second commonest, and z axis – the third commonest response, from medical students on that question (in blue). For VML model (in green), the x axis is the prediction with highest probability, y axis – second highest and z-axis, third highest probability. The numbers 0 to 9 on each axis represent the 10 categories of tissue: 'cerebellum', 'cerebrum', 'heart', 'kidney', 'liver', 'lung', 'pancreas', 'skin', 'stomach', & 'trachea' . The quiz consisted of 100 questions (*n *= 100) (Figure generated by Matplotlib python library, version 3.3.4, https://matplotlib.org/).

A plot of top 3 commonest answers, per question basis, from both MS and VML, shows a diffuse scatter. No clustering is noted between the top 3 answers/probabilities of MS and VML, on an image-by-image basis (Fig. 12).

The IML model was decisive in its predictions in majority of the cases: only 02 questions out of 100 in the quiz set were answered equivocally, i.e. where the prediction with the 2nd highest probability was more than half of the first. Thus the IML model was not comparable to MS regarding equivocal responses.

#### Images that were difficult to classify, for both MS and VML

**Figure 13:**
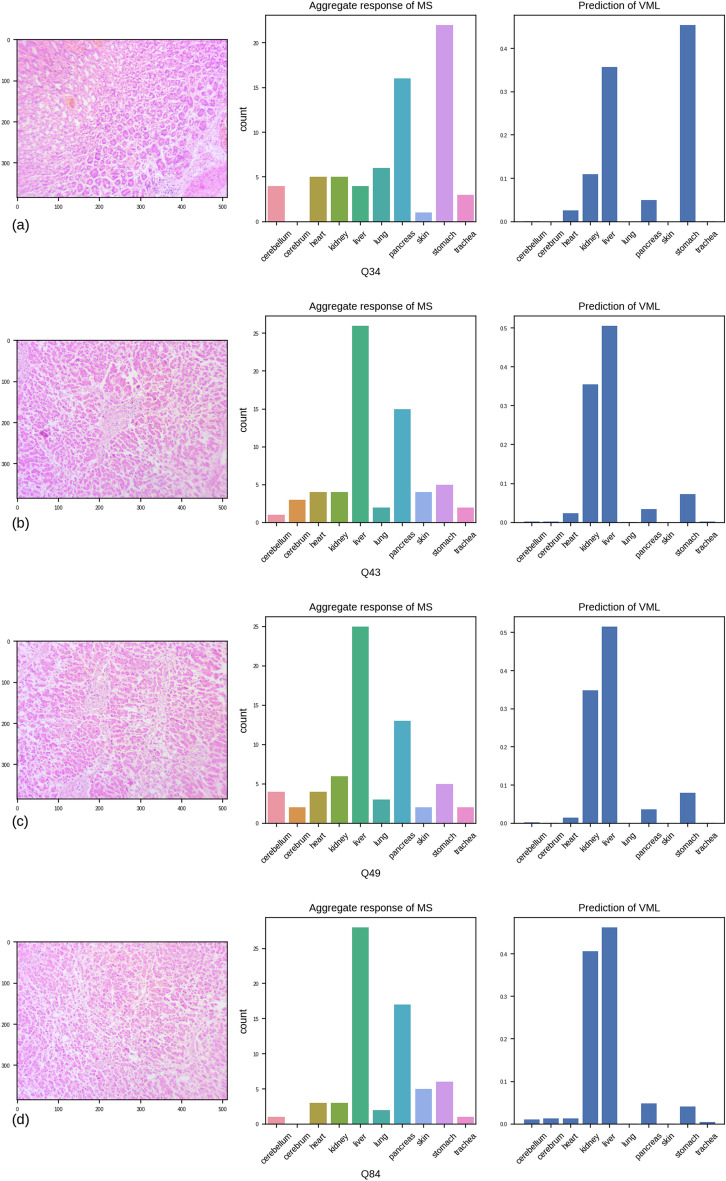
The four images in the quiz set where both medical students and VML model have produced mixed responses, with the 2nd commonest response having more than half the probability/ frequency of the commonest response. (**a**) stomach, (**b**), (**c**) & (**d**) liver. (Figure generated by Matplotlib python library, version 3.3.4, https://matplotlib.org/).

**Figure 14:**
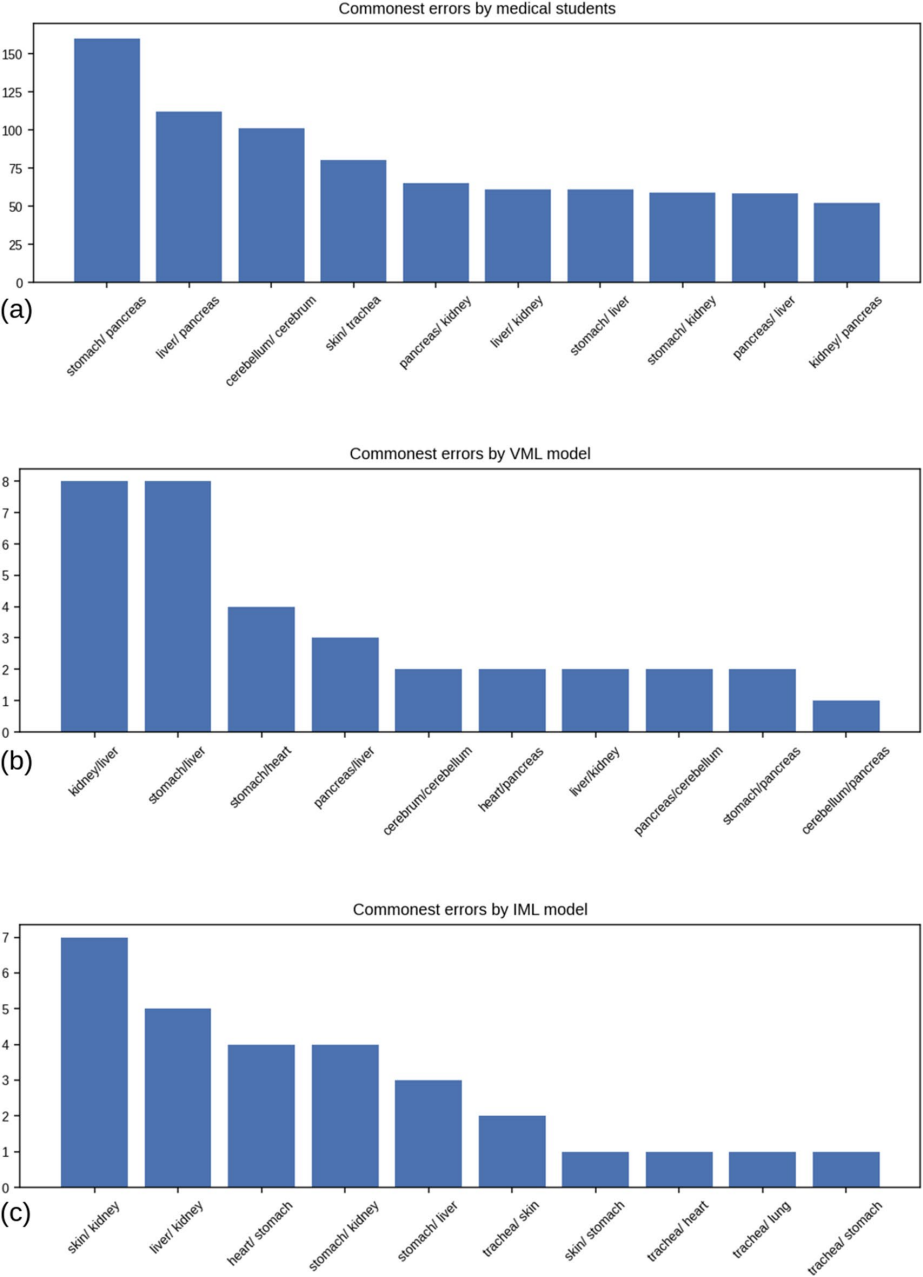
Error patterns of MS, VML & IML: (**a**) commonest errors of the medical students (MS) over the quiz set (n = 6557); (**b**) commonest errors of VML on the validation dataset (n = 300); (**c**) commonest errors of the IML on validation dataset (*n* = 300); a label such as ‘liver/ kidney’ indicates that ‘liver’ was mistaken for ‘kidney’ (Figure generated by Matplotlib python library, version 3.3.4, https://matplotlib.org/).

We found only 04 (four) images where *both* MS and VML have produced a grossly mixed response, i.e. the frequency (probability) of the second choice was more than half of that of the first choice. The second choices of MS and VML were different in all such images (Fig. 14). Three of these images belong to the class ‘liver’. While both MS and VML model have generated correct classification with the greatest probability, it is interesting to note that the **2nd most frequent answer from MS was always ‘pancreas’, and the 2nd highest probability from VML model was always ‘kidney’**. ‘Stomach’ is the 3rd most probable prediction in all three images, by both MS and VML (Fig. 13).

### Comparison of MS on quiz questionnaire and VML/IML on full validation set

The ten commonest errors in classification by aggregate of MS, as compared to VML model (the results of the VML model on the full validation set of 300 images, not just the quiz images) & the IML model (on the full validation set), are shown in Table 10:

**Table 10:** Commonest errors of MS, VML & IML model; 66 medical students have provided 6557 responses in a quiz questionnaire of 100 images; the VML and IML model were evaluated on the full validation set (*n* = 300).

MS (n = 6557 responses)				VML (*n* = 300)				IML (*n* = 300)			
Original	Predicted	Number of errors	Proportion among all errors	Original	Predicted	Number of errors	Proportion among all errors	Original	Predicted	Number of errors	Proportion among all errors
Stomach	Pancreas	160	19.78%	Kidney	Liver	8	23.53%	Skin	Kidney	7	24.14%
Liver	Pancreas	112	13.84%	Stomach	Liver	8	23.53%	Liver	Kidney	5	17.24%
Cerebellum	Cerebrum	101	12.48%	Stomach	Heart	4	11.76%	Heart	Stomach	4	13.79%
Skin	Trachea	80	9.89%	Pancreas	Liver	3	8.82%	Stomach	Kidney	4	13.79%
Pancreas	Kidney	65	8.03%	Cerebrum	cerebellum	2	5.88%	Stomach	Liver	3	10.34%
Liver	Kidney	61	7.54%	Heart	Pancreas	2	5.88%	Trachea	Skin	2	6.90%
Stomach	Liver	61	7.54%	Liver	Kidney	2	5.88%	Skin	Stomach	1	3.45%
Stomach	Kidney	59	7.29%	Pancreas	Cere-bellum	2	5.88%	Trachea	Heart	1	3.45%
Pancreas	Liver	58	7.17%	Stomach	Pancreas	2	5.88%	Trachea	Lung	1	3.45%
Kidney	Pancreas	52	6.43%	cerebellum	Pancreas	1	2.94%	Trachea	Stomach	1	3.45%
Total		809				34		Total		29	

The ‘stomach’ class accounted for the highest number of errors in both VML and MS, producing 34.84% of all errors by MS and 41.17% of all errors by VML model. The commonest error by medical students was misclassifying stomach as pancreas (160 errors), liver as pancreas (112 errors), and cerebellum as cerebrum (101 errors) (Fig. 14).

The commonest error of the VML model on full validation set were misclassifying kidney as liver (08 errors), misclassifying stomach as liver (08) & misclassifying stomach as heart (04). There is no overlap between the commonest errors of MS and VML model (Fig. 14). The pattern of errors of the IML model is distinct from both MS & VML; ‘skin’ is the commonest misidentified class, followed by liver and heart (Fig. 14).

### Comparison with students with scores close to ML models

The overall accuracy of MS was much lower than the VML model/IML model, and thus their confusion matrices were not directly comparable. We selected 05 students whose accuracy in quiz was in the range 0.90–0.92, i.e. close to that of the VML model. We constructed their aggregated error matrix (Table 11).

**Table 11:** Aggregated confusion matrix of 05 students who attained accuracy close to the VML model.

Predicted	Cere-bellum	Cere-brum	Heart	Kidney	Liver	Lung	Pancreas	Skin	Stomach	Trachea
**Actual**										
Cere-bellum	54	6	0	0	0	0	0	0	0	0
Cerebrum	0	60	0	0	0	0	0	0	0	0
Heart	0	0	59	0	0	0	1	0	0	0
Kidney	0	0	0	58	0	0	0	0	2	0
Liver	0	0	0	1	47	0	10	0	2	0
Lung	0	0	0	0	2	57	0	1	0	0
Pancreas	0	0	0	0	3	0	54	0	3	0
Skin	1	1	0	0	0	0	0	57	0	1
Stomach	0	0	0	1	0	0	18	0	41	0
Trachea	0	0	0	0	0	0	0	1	0	59

We then compared this confusion matrix with the error matrix of the VML and IML model over the entire validation set (Table 3). A Kolmogorov–Smirnov test showed K-S statistic 0.1, *p*-value = 0.702, suggesting similar error profiles between the VML/IML and this subset of medical students.

### Results of OTS images

The results of MS and VML on OTS images (10 images) are as follows. A ‘likeness score’ has been assigned based on resemblance of original and predicted tissue, i.e. if ‘salivary gland’ is predicted to be ‘pancreas’, a likeness score of 01 is allotted (Table 12).

**Table 12:** Performance of MS, VML & IML model on out-of-training-scope images.

Original	VML prediction	Likeness score (VML)	Commonest answer by MS (*n* = 44)	Likeness score (MS)	IML prediction	Likeness score (IML)
Parathyroid	Stomach	0	Liver	0	Lung	0
Cervix	Skin	1	Skin	1	Skin	1
Intestine	Lung	0	Stomach	1	Stomach	1
Intestine	Lung	0	Stomach	1	Lung	0
Parathyroid	Lung	0	Liver & lung	0	Lung	0
Intestine	Lung	0	Stomach	1	Lung	0
Salivary gland	Lung	0	Pancreas	1	Lung	0
Endometrium	Lung	0	Pancreas	0	Lung	0
Salivary gland	Lung	0	Lung	0	Lung	0
Cervix	Skin	1	Skin	1	Lung	0
Total		2		6		2

**Figure 15:**
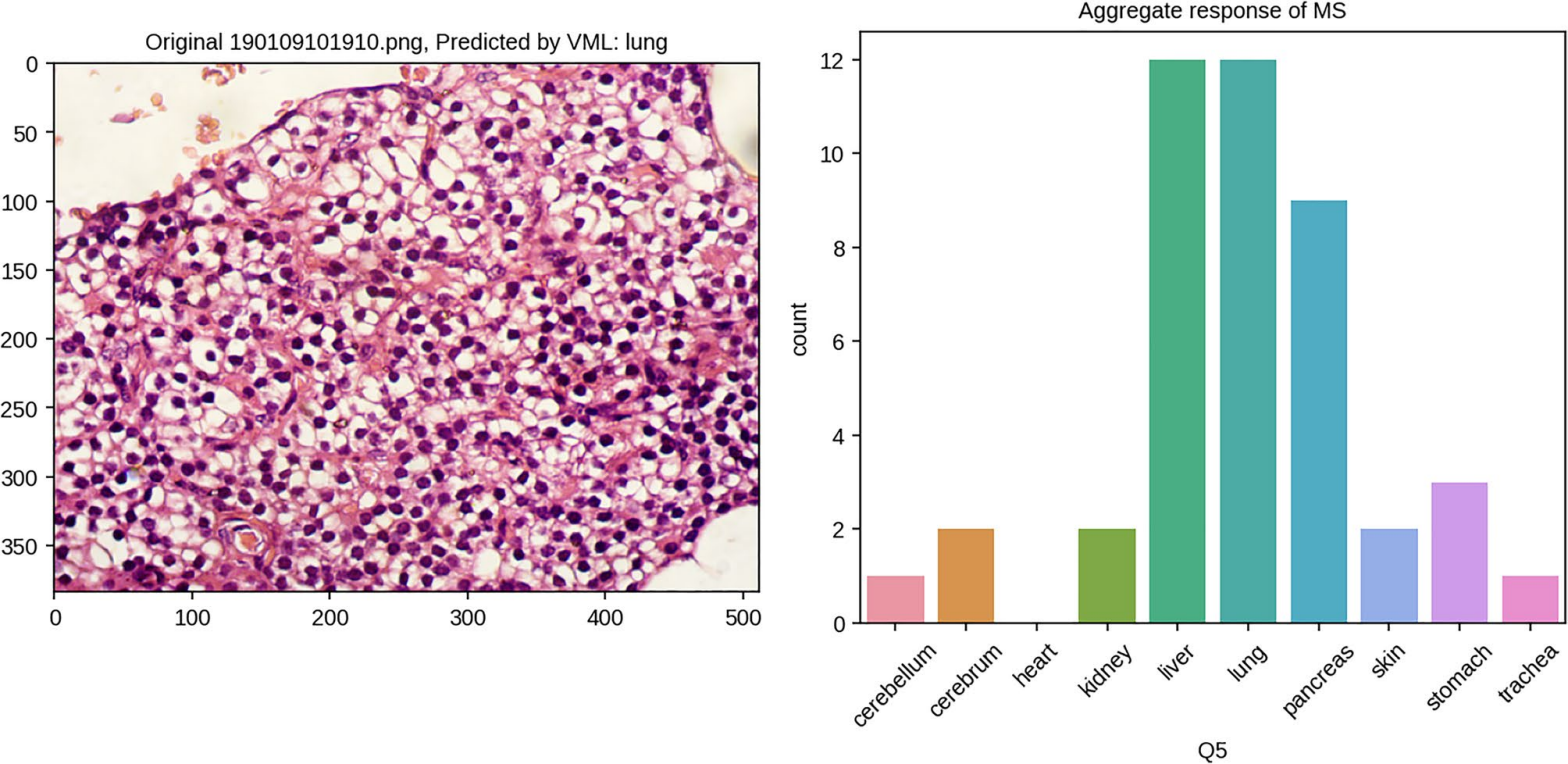
Parathyroid tissue predicted by VML & IML to be ‘lung'; the opinion of medical students is split between several classes (Figure generated by Matplotlib python library, version 3.3.4, https://matplotlib.org/).

The same set of images was shown to the MS and their responses recorded. As shown in the table, the VML & IML model have clearly overfitted on the class ‘lung’. This is concordant with the perfect accuracy (100%) of both VML & IML in recognising lung tissue in the validation set. Unlike the VML model, majority of medical students have been able to place the images in the category which it resembles histologically, i.e. they have consistently classified intestine as ‘stomach’ (possibly, they could recognise intestine, but selected ‘stomach’ because the options were limited to only ten classes of the training dataset). In recognising ‘parathyroid’ and ‘endometrium’, MS, VML & IML model have produced random results (Fig. 15); this is expected – as the VML/IML model was never trained on these tissues. Also, the medical students were not trained on these tissues (parathyroid, endometrium).

## Discussion

The emergence of DNNs has rekindled academic interest in the true nature of perception and working of the mind. Qualities that were erstwhile known to be decidedly biological, such as visual recognition of objects, have been successfully reproduced in DNN models. Even the most abstract of faculties, mathematics and symbolic logic, have been implemented with DNNs with some degree of success^52^. However, a direct comparison of DNN with human visual recognition is difficult because of several reasons. Humans, although well trained by a lifetime of experiences, are prone to the occasional error in visual recognition (such as optical illusions, wrong interpretation of color, even misrecognition of faces^53^). Adversarial images, which can confound DNN models, have also been shown to affect humans when limited by time for decision-making^54^.

In early studies on DNNs, the performance of humans was assumed to be 100% accurate, i.e. human visual recognition was the gold standard against which DNN models were compared. However, it was soon realised that in specialised domains (such as recognising dog breeds), humans were just as naive as machines, and DNN models may outperform humans^55^. But even in such studies, the base class ‘dog’ is not outside the purview of human experience, although the subclasses (breeds) might be unknown to the human subjects. Thus, there still remains a bias in favor of humans.

Funke et al., in their guideline, describe the control variables when comparing human and ML performance on image recognition^56^. One such control is ‘aligning experimental conditions for both systems’, i.e. the matching operation. They mention that ‘the human brain profits from lifelong experience, whereas a machine algorithm is usually limited to learning from specific stimuli of a particular task and setting.’ The same point was highlighted by Cowley et al. in a recent paper on designing a framework for human–machine learning comparison. They observed that while the ML model can be trained only with a limited set of data, the same does not apply to humans and it was not possible “ … to implement a one-shot learning task in human participants using natural images and categories that humans already have experience with” ^57^. We have tried to remove the bias of experience, by studying an image dataset which is novel to both human and machines (i.e. histological images).

However, it is to be emphasised that the training period differed between humans and machines, in our study. This is due to the differences in machine and human learning: we have selected medical students who had already been taught histology as part of their curriculum in first year of medical school. This is because humans need a minimum period of training to gain proficiency, and to develop pattern recognition skills which they can generalise to novel problems. An ML model is much faster to train (typically, within hours or days); humans cannot be expected to learn anything within such a short period.

### Pattern of errors in MS & VML/ IML model

Overall, the pattern of errors of medical students and VML/IML model is distinctly different, as seen by the overall kappa and chi-squared Goodness of Fit tests (Table 9). In the quiz questionnaire, there were only 04 images which generated mixed response *both* from MS and VML. However, the pattern of the responses (i.e. 2nd and 3rd common response) was different in MS and VML. Whereas the commonest error by MS was stomach/ pancreas (i.e. classifying stomach as pancreas) and kidney/pancreas, the commonest errors of the VML model was kidney/liver and stomach/ liver. This suggests ‘overfitting’ on pancreas by MS, and on liver by VML model.

Interestingly, the images of ‘stomach’ class were obtained from gastric biopsies, and were thus missing muscularis propria. Thus, the ‘stomach’ class has assisted us to uncover hidden bias in both MS and VML. The medical students tend to mistake the stomach for pancreas, whereas the VML model frequently mistook the stomach for liver.

The IML model, however, is much more certain in its responses (only 2% equivocal responses in the quiz set). It shows a bias for the ‘kidney’ class, and has frequently mistaken skin and liver for kidney. This pattern is also different from medical students. Because of its higher accuracy and greater certainty than VML, we felt that the IML model was less suitable for comparison with medical students.

### Visualising deeper layers of the DNN in misclassified images

**Figure 16:**
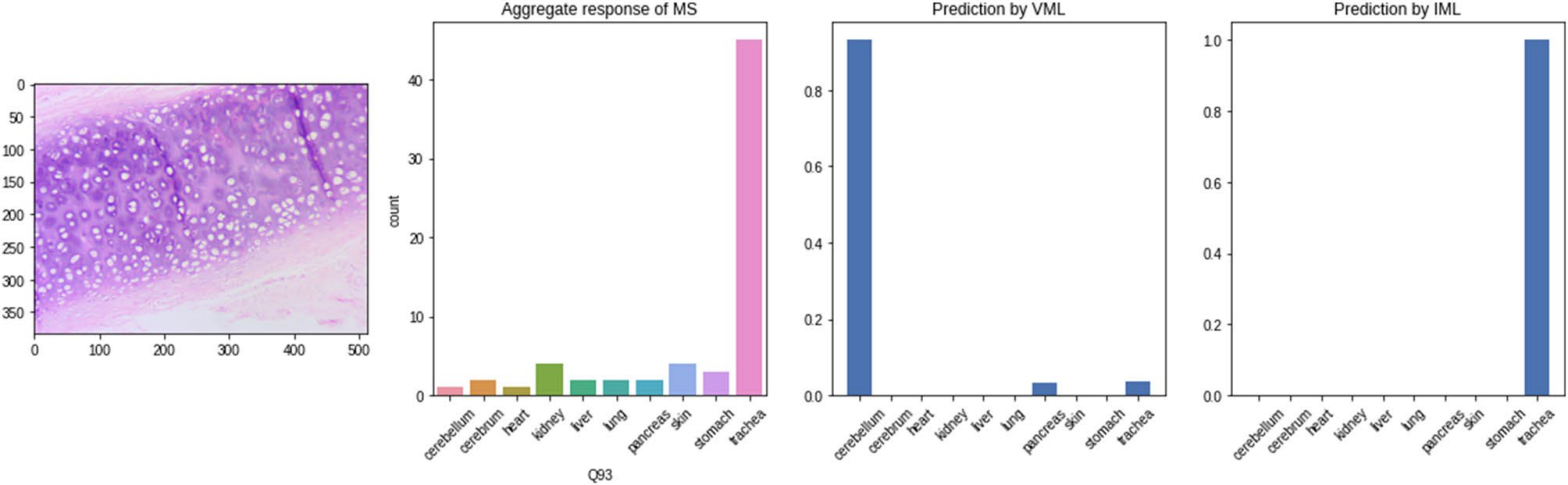
An image of trachea recognised correctly by majority of MS, misclassified by VML and correctly classified by IML (Figure generated by Matplotlib python library, version 3.3.4, https://matplotlib.org/).

**Figure 17:**
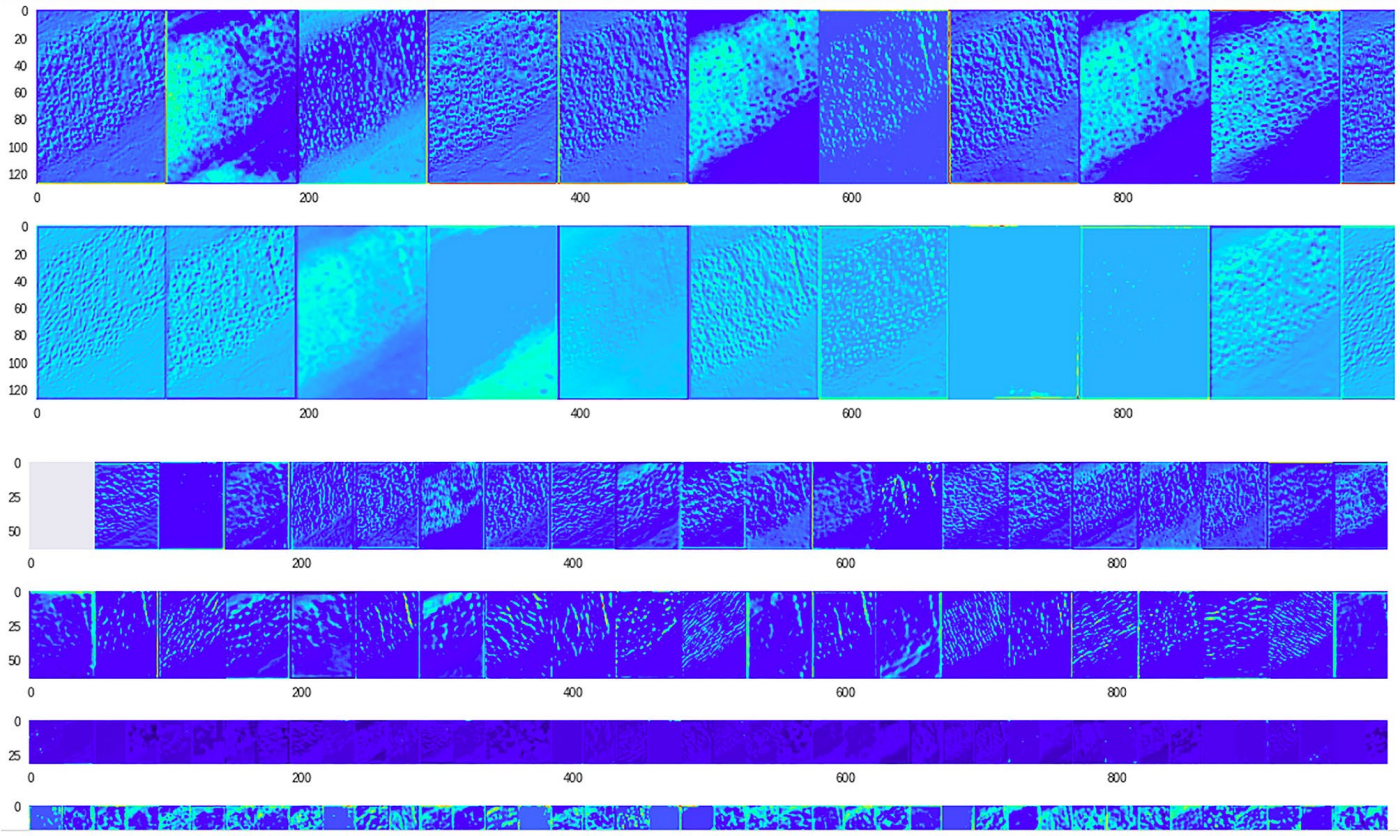
The VML model processing the image from 'trachea' class (Fig. 16), eventually misclassifying it as ‘cerebellum’. The inner layers (convolution layers only) are represented here. The thick band of cartilaginous tissue is the prominent feature of this image, and has persisted till the last convolution layers. (For visualisation purposes, a psuedocolor scheme has been used to render the deeper layers, and only the first few slices of initial convolution layers are shown) (Figure generated by Matplotlib python library, version 3.3.4, https://matplotlib.org/).

Following is a serial visualisation of convolutional layers of the VML, which is processing an image of trachea, and misclassifies it as cerebellum. The MS group have however, correctly recognised this image as trachea with a clear majority (Figs. 16 and 17).

**Figure 18:**
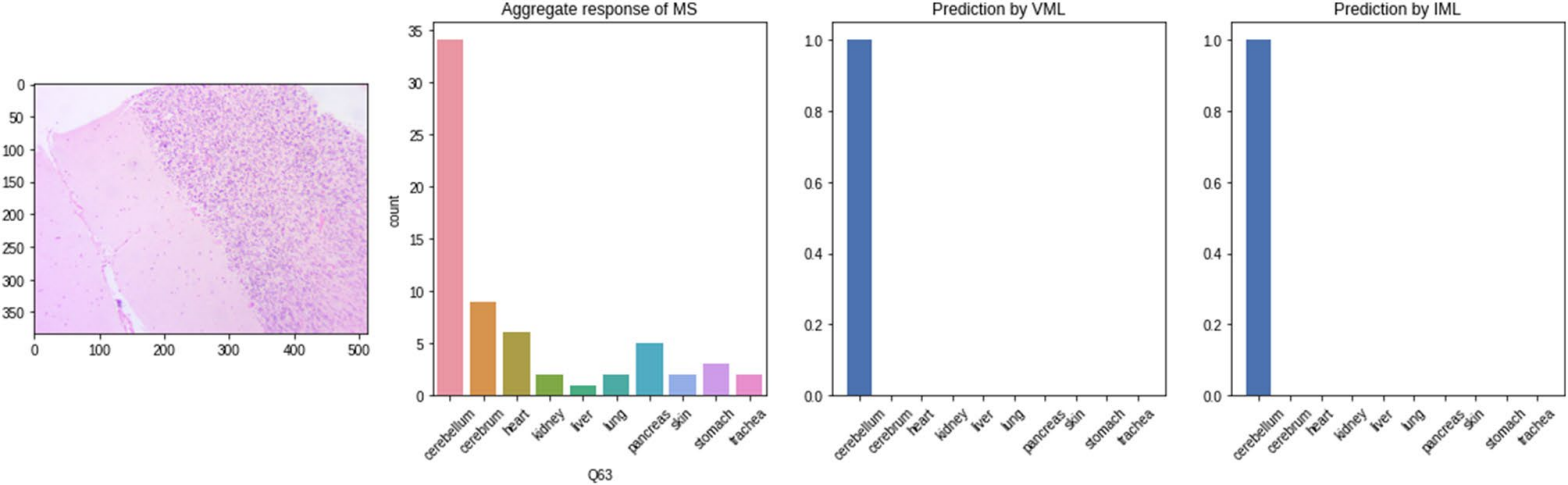
An image of cerebellum correctly classified by majority of MS, VML and IML (Figure generated by Matplotlib python library, version 3.3.4, https://matplotlib.org/).

**Figure 19:**
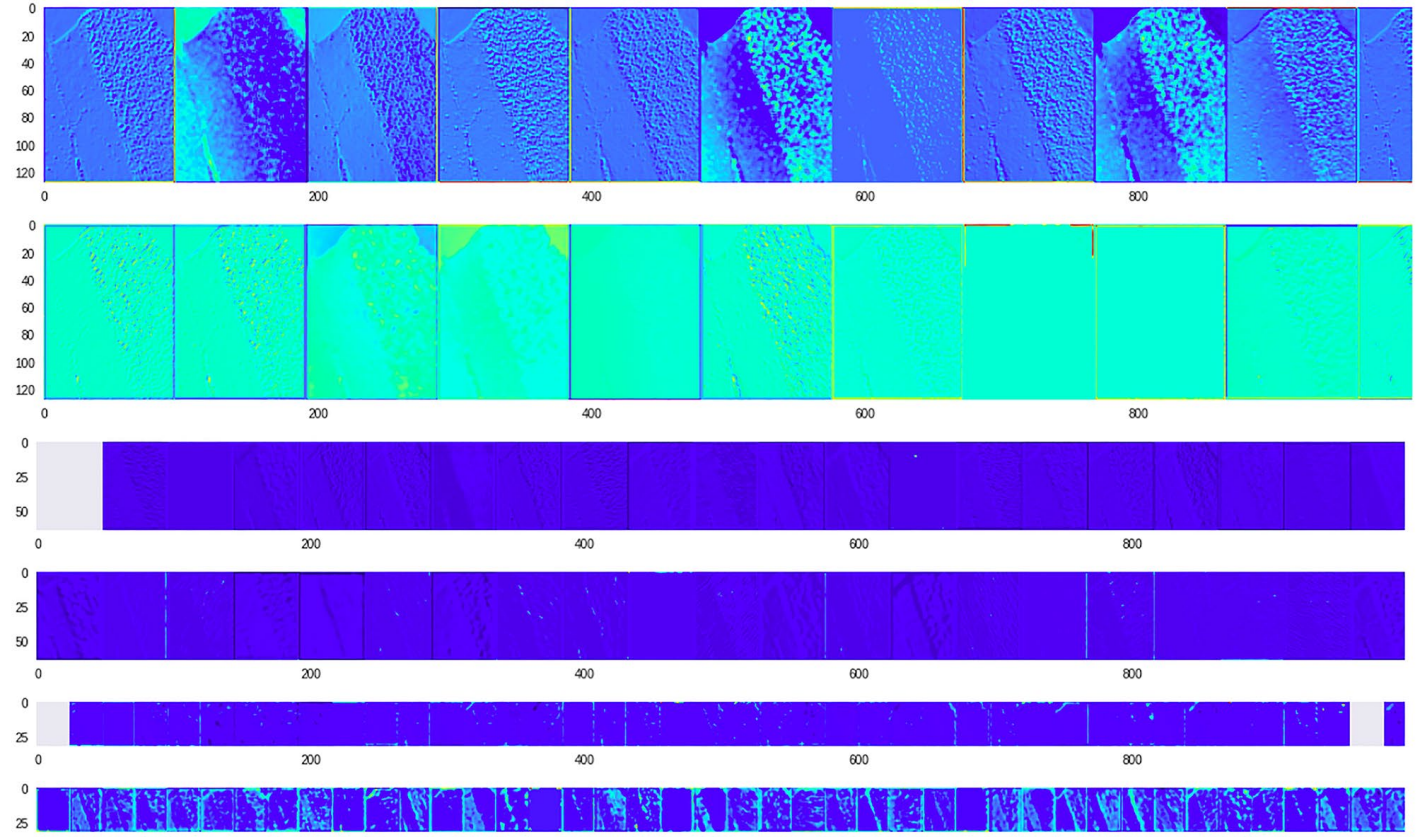
Image of 'cerebellum' class correctly classified by the VML; there is similarity of the initial first 10 slices of the first convolution layer, with the previous figure (Fig. 18). The granular layer has persisted till the last convolution layers. (Figure generated by Matplotlib python library, version 3.3.4, https://matplotlib.org/).

We compared this with an image of ‘cerebellum’ class that was correctly classified by the VML. The similarities in the convolutional layers are evident. It is possible that the cartilaginous band in trachea led to the misclassification of the previous image as cerebellum, due to similarities with the granular layer of cerebellum (Figs. 18 and 19).

**Figure 20:**
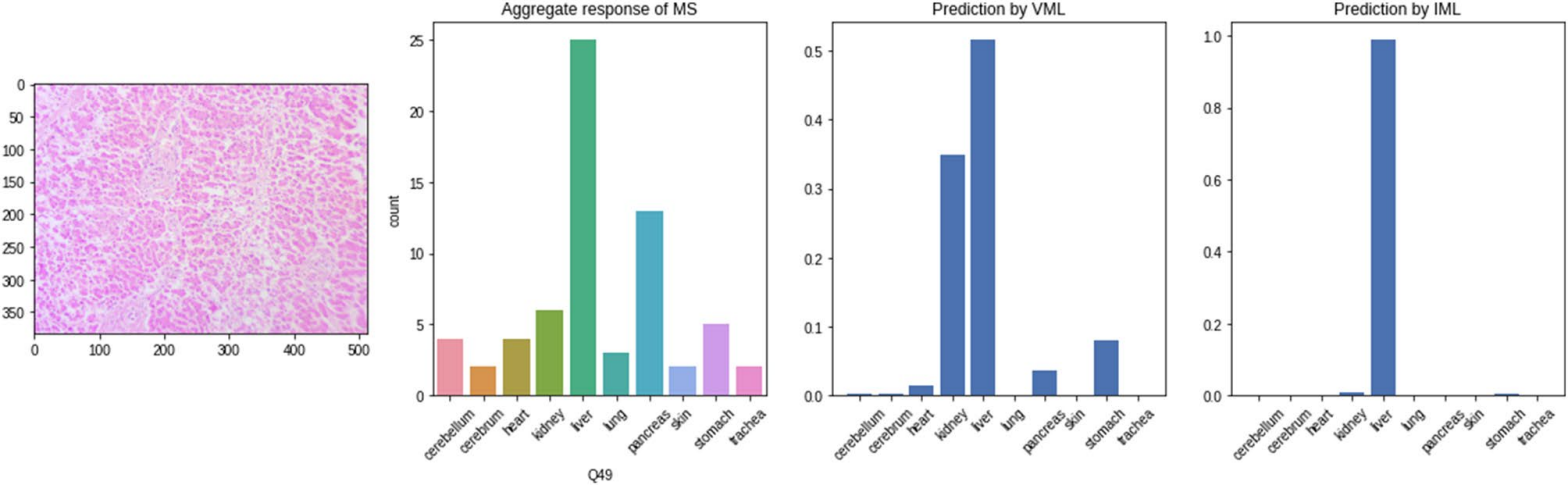
Image from ‘liver’ class producing mixed response from MS & VML but unequivocal response from IML (Figure generated by Matplotlib python library, version 3.3.4, https://matplotlib.org/).

This is in stark contrast to an image from ‘liver’ class, which was recognised by most of the students, and also by VML model with a mixed response (Fig. 20). The IML model, however, unequivocally classifies this as ‘liver’.

**Figure 21:**
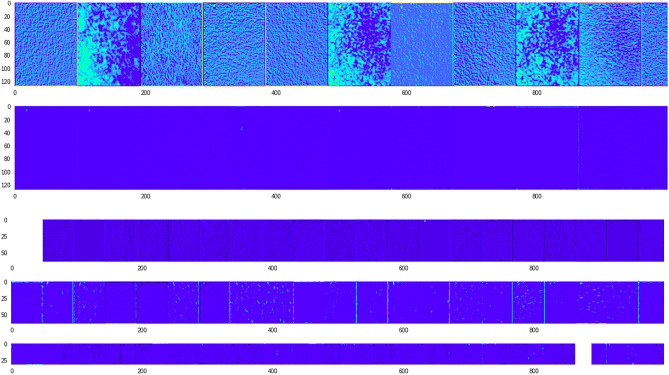
Convolution layers of the image in previous figure (Fig. 20) within the VML. No specific identifying feature has persisted till the last few convolution layers (Figure generated by Matplotlib python library, version 3.3.4, https://matplotlib.org/).

Deeper layers of the VML reveal specific signatures of this image which do not persist till the innermost layers (Fig. 21).

**Figure 22:**
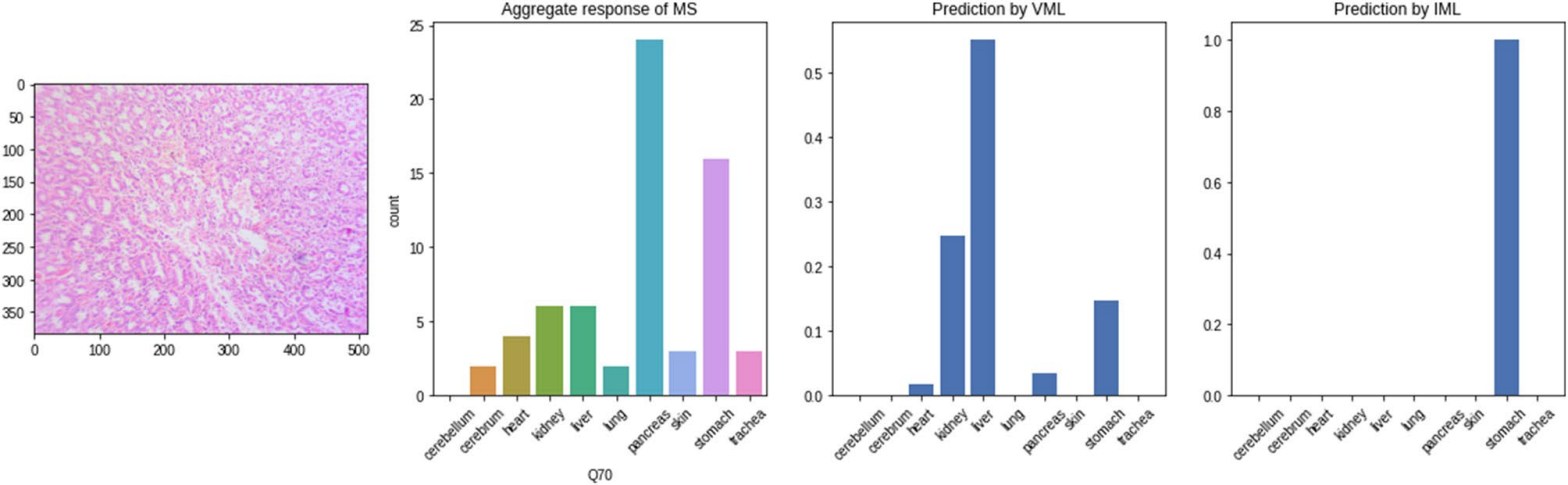
Stomach mucosa producing mixed response from MS, wrongly identified by VML and correctly identified by IML (Figure generated by Matplotlib python library, version 3.3.4, https://matplotlib.org/).

**Figure 23:**
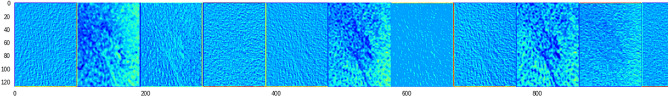
The artifactual linear blank space in the previous figure (Fig. 22) has prominently featured in the first few convolutional layers of VML model (Figure generated by Matplotlib python library, version 3.3.4, https://matplotlib.org/).

The ‘stomach’ tissue, which has turned out to be the most confounding in both humans and VML model, was mistaken for all other classes of tissue. Figure 22 shows stomach tissue mislabeled as liver by the VML model. The first few convolutional layers show the artifactual blank space which has given the gastric mucosa a vague lobular appearance. (Fig. 23).

### Comparison of errors of MS and ML models

**Figure 24:**
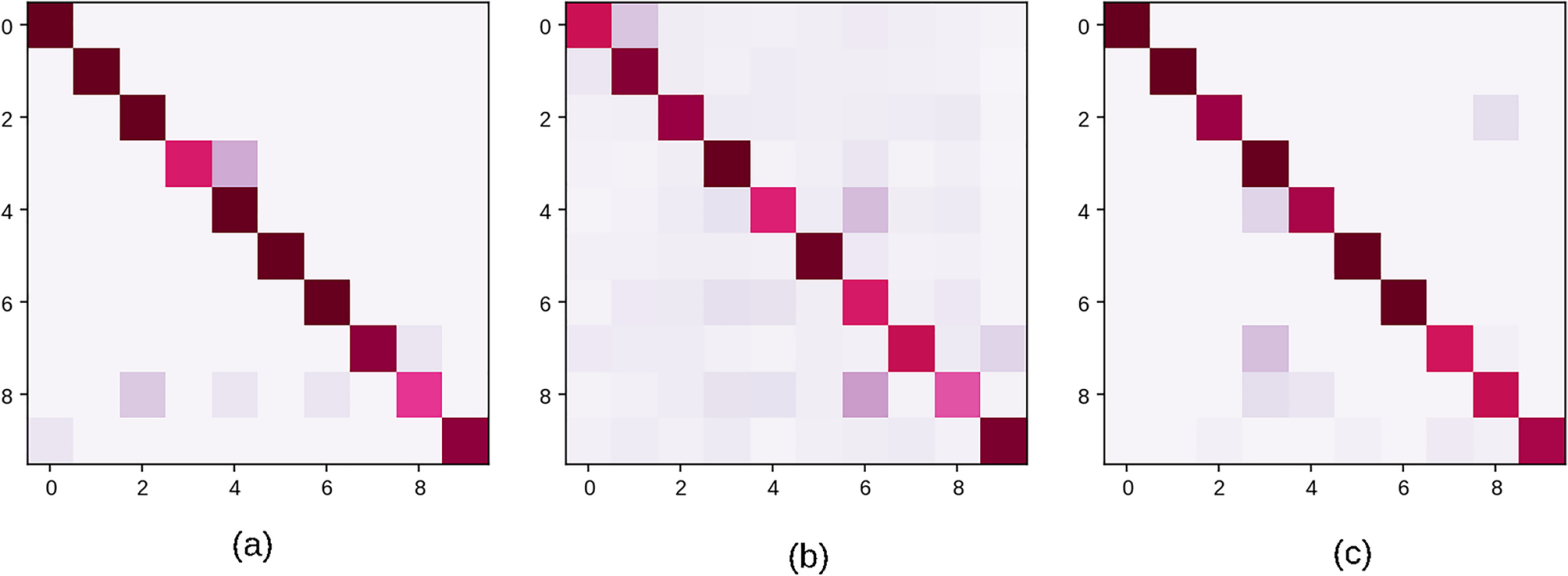
Error matrices of VML, MS and IML: (**a**) Confusion matrix of VML over the entire validation set (*n* = 300); the dark spots along the diagonal represent correct answers; (**b**) Aggregated confusion matrix of medical students over the quiz images (*n* = 6557); dark spots are noted outside the diagonal line, representing wrong answers; (**c**) Confusion matrix of IML model on validation set (*n* = 300); the IML is more accurate, and shows less equivocal predictions, than the VML model (Figure generated by Matplotlib python library, version 3.3.4, https://matplotlib.org/).

An interesting finding in the study was that students who have scores close to the VML/ IML model (i.e. between 0.9–0.92), **produce similar error profile of the VML/IML model** (when the performance of VML/IML model over entire validation set is considered). A Kolmogorov–Smirnov test failed to reject the null hypothesis (p = 0.702)^58^. However, when compared with *all* medical students, the pattern of errors was distinctly different. (Fig. 24).

**Figure 25:**
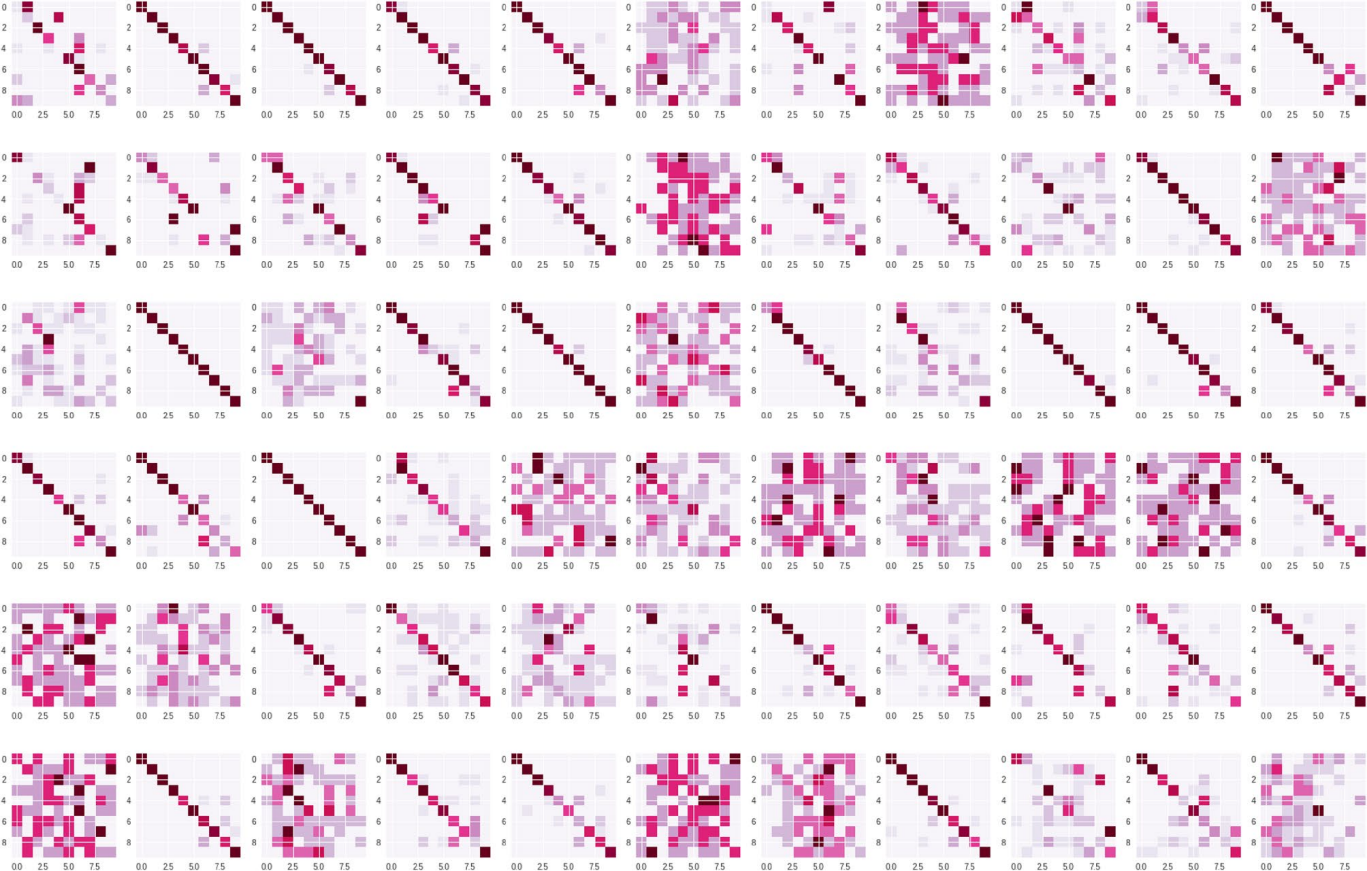
Confusion matrix of all 66 students over the quiz set; the overall error profile is very dissimilar to ML models; only the best performing students produced a similar error profile as that of the VML model (Figure generated by Matplotlib python library, version 3.3.4, https://matplotlib.org/).

In a study by Dodge et al. on image dataset of different dog breeds, not only did humans outperform machines on serially distorted images, but the pattern of errors was also significantly different between humans and machines;^12^ the authors went on to suggest that the inner representation of images vary between humans and machines. However, the accuracy of the DNN model in their study was much lower than humans. In the present study, very few students have reached accuracy close to the machine (Fig. 25); between these few students and VML/ IML model, the error profile was similar.

The IML model, however, in addition to being more accurate than both MS & VML, also shows a distinct error profile (with a bias for the ‘kidney’ class). The IML model seems to be more ‘certain’ in its decision than VML model and aggregate of medical students. Whereas the aggregate of medical students provided equivocal answers to 12 questions (12%) of the quiz set, and the VML produced equivocal predictions in 20 questions (20%), the IML generated an equivocal prediction in only 02 questions (2%). (By ‘equivocal’, we mean where the second commonest answer/prediction was ≥ 50% of the commonest answer/prediction–in frequency).

### Likeness score of MS, VML & IML model in OTS images

In the OTS image set, we created a metric ‘likeness score’ based on histologic similarity of actual and predicted classification: if ‘cervix’ was recognised as ‘skin’, a likeness score of 01 was awarded (owing to presence of squamous epithelium in both of the tissues). The medical students (total likeness score = 6) outperformed the VML/IML models (total likeness score = 2). This was due to overfitting of the ML models on the ‘lung’ class (07 & 08 of the 10 images in the OTS set was classified by VML and IML model as ‘lung’, respectively), as well as indicating the **higher efficacy of humans in pattern recognition from OTS images** (i.e. finding similarities of architecture between ‘salivary gland’ and ‘pancreas’, both of which are exocrine glands).

The failure of the VML in recognising ‘likeness’ between tissues hints at the same problem faced by Funke et al. while trying to teach DNN models the concept of ‘closed’ and ‘open’ shapes;^34^ they realised that even after intensive training, the DNN model did not learn the *concept* of a closed shape. In our study, the overfitting on ‘lung’ class suggests that while the VML/IML model can recognise lung and pancreas with reasonable accuracy, it has not learnt the concept of a glandular structure, as evident by its misclassification of ‘salivary gland’ as ‘lung’.

### Comparison with previous studies

Several efforts have been made in the recent past, to compare human and machine learning in the field of image recognition. The design of these studies varies in their problem space and models used, and are thus not directly comparable to the present study. (Table 13) However, our conclusions are similar to the studies by Kuhl 2020, Dodge 2017 and Fleuret 2011: that humans generalise to a larger problem space much faster than ML models, as evident by performance of medical students on the OTS image data.

**Table 13:** Summary of recent studies comparing human and machine learning, along with the approach taken in the present study.

Author	Problem space	ML model	Population for comparison	Conclusion
Orosz et al.^59^	Annotating text (legal documents) for features specific to case instance	Support vector machine	Laymen, lawyers and legal editors	ML model matches accuracy of legal editors
K¨uhl et al.^60^	Inferring rules from simple 3 × 3 grid patterns	Logistic regression, decision tree, neural network	General population	Humans learn more from small training set than machines; large differences seen in learning curves of ML models depending on pattern and rule
Dodge et al.^12^	Labeling breeds of dogs from normal and distorted images of dogs	Modified VGG16, InceptionV3 and ResNet50 (transfer learning)	General population	Humans score better accuracy on distorted images than ML models; there is no correlation between the errors of humans and ML models
Fleuret et al.^11^	Inferring rules from artificially generated shapes	Adaboost, Support vector machine	General population	Humans infer rules with better accuracy from a much smaller number of examples than ML models
Present study	Classification of histological images into defined classes of tissue	Convolutional neural network	Medical students	Within scope of training, ML model performed better than students. However, students were able to extend their learning to new domains

## Limitations

The study was limited by the quality of tissue preservation: in particular, the images from ‘liver’ and ‘kidney’ class were retrieved from autopsy specimens and some distortion of tissue architecture was present in few of the images. The images from ‘stomach’ class were from mucosal biopsies, resulting in loss of orientation of tissue. It must be mentioned that the ‘stomach’ class of tissue has, inadvertently, uncovered hidden bias in both MS and VML model, as mentioned in results.

Again, the medical students represent variable amounts of prior knowledge of histology, as is expected in any group humans with diverse academic aptitudes. This has reflected in the variation in accuracy of medical students. It is difficult to correctly match the exact amount of ‘training’ between humans and machines: we have selected medical students with some prior training in histology, i.e. trained for 2 months in histology as part of their curriculum in first year of medical school. This is because an initial, informal assessment on completely naive students had shown that without a period of training of at least a month, human students did not achieve > 10% accuracy in recognising 10 different classes of tissue. Certainly, the time taken by the VML/IML model to train on these tissues (2 h) is grossly insufficient for human students.

The quiz was conducted without any time limits, and answering each question was not mandatory (unanswered questions were excluded from final analysis). With the didactic experience of three of the authors, the psychology of students when facing a quiz was taken into consideration. We thus felt that making questions mandatory, or putting a time limit, would force the students to cause mistakes. It was decided not to implement artificial restrictions on human students. It is at this point that ML models significantly differ from medical students: an ML model, given an image, will *always* produce an answer; it cannot ‘pass’ questions. This is a fundamental limitation in comparing human and machine learning. However, we decided to mitigate this limitation by excluding unanswered questions (from medical students). In the course of analysis, especially when calculating Cohen’s kappa, unanswered questions by students were placed into the ‘other’ category and counted as errors. It must also be noted that even without making an answer mandatory, 6557 responses were received from medical students out of a possible 6600 (99.34%).

## Conclusion

The present study compares the performance of two deep learning models (a modified VGG16 model and a modified Inception model) with a group of human medical students, on an image dataset which is novel to both: the deep learning models and humans have had no prior exposure to histological images. Our findings suggest that within the scope of training, the deep learning models perform better than 80% medical students. The medical students (MS) and VGG16 model (VML) faced similar difficulties in classification, as evident by the fact that ‘stomach’ was most difficult to classify by both of them, although the error profile–what they mistake it for–was different between MS and VML; in contrast, the Inception model (IML) was most frequently confounded by the ‘skin’ class. However, the students who have reached accuracies close to the VML/IML, also tend to replicate the same pattern of errors in image recognition as that of the ML models. This suggests a degree of similarity between how an ML model and humans extract features from an image when their accuracies are similar.

If asked to classify images outside the scope of training, humans perform better at recognising patterns and likeness between tissues. We suggest that ‘training’ (in the context of machine learning) is not the same as ‘learning’, and humans can extend their pattern-based learning to different domains outside of the training set.

Link to training notebook (annotated source code) of the DNN models, the VGG16 and Inception models (in H5 format) is provided in Data Availability section.

## Future directions

The present study offers an insight into the inner representation of visual information, of human and machine learners, on a novel dataset. The study can be extended to any direction, particularly with state of-the-art-image classification models such as Xception and Vision Transformers. It would also be interesting to reproduce this study with altered or fragmented images, to assess performance of both the learners in recognising local features from an image. Such studies will bring the differences between humans and machine learning models, in the context of image recognition, in sharper focus.

## Data Availability

Quiz questionnaire with images: https://forms.office.com/Pages/ResponsePage.aspx?id=DQSIkWdsW0yxEjajBLZtrQAAAAAAAAAAAAe__YDJd8ZUMUJDU1JOUjJBUzBISDk5UDJaRkIwUjhITi4u. OTS questionnaire with images: https://forms.office.com/Pages/ResponsePage.aspx?id=DQSIkWdsW0yxEjajBLZtrQAAAAAAAAAAAAe__YDJd8ZUMU9IVVNWRUtRU0I2MEMyMUFLTExZVkhXTC4u. Annotated source code of the DNN models, the responses from students on quiz and OTS images are uploaded at: https://github.com/cmacus/histo_ml_human. Any other data pertaining to the study will be made available on request.
